# A genetic interaction between *DED1* and *HAT1* in *Saccharomyces cerevisiae* reveals a role for Hat1p in cytoplasmic RNA granule accumulation

**DOI:** 10.1093/g3journal/jkaf307

**Published:** 2025-12-19

**Authors:** Audrey J Panko, Aidan Winters, Nicholas R Rothbard, Angela K Hilliker

**Affiliations:** Department of Biology, University of Richmond, 138 UR Drive, Richmond, VA 23173, United States; Department of Biology, University of Richmond, 138 UR Drive, Richmond, VA 23173, United States; Department of Biology, University of Richmond, 138 UR Drive, Richmond, VA 23173, United States; Department of Biology, University of Richmond, 138 UR Drive, Richmond, VA 23173, United States

**Keywords:** translation regulation, acetyltransferase, RNA helicase, stationary phase, cytoplasmic RNA granules

## Abstract

Ded1p is an essential translation initiation factor that interacts with and is modulated by eIF4F. Ded1p also promotes the assembly and disassembly of stress granules, which contain nontranslating mRNAs and translation initiation factors. As Ded1p affects both mRNA storage and translation initiation, regulation of Ded1p's function may affect whether mRNAs are translated or localized to cytoplasmic RNA granules. To identify regulators of Ded1p in *Saccharomyces cerevisiae*, we screened for overexpression suppressors of the severe growth defect conferred by high levels of Ded1p. We found that overexpression of *HAT1*, a lysine acetyltransferase, can suppress the growth defect conferred by overexpression of *DED1*, but we do not find evidence of direct acetylation. We demonstrate that *HAT1* antagonizes the accumulation of P-bodies under short-term stresses. Under sustained glucose deprivation during the stationary phase, strains lacking *HAT1* form more stress granules and have a survival advantage. Given the genetic connection between *HAT1* and *DED1*, we screened for other lysine acetyltransferases and deacetylases that have a genetic interaction with *DED1*, identifying several more of these post-translational modifiers as possible regulators of mRNA storage and/or translation. These results demonstrate connections between acetylation and the control of cytoplasmic mRNA localization.

## Introduction

Translation is highly regulated to control protein production and is one of the most energy-intensive processes in the cell. Changes in cellular health and energy status are communicated via signaling pathways to alter translation efficiency (reviewed in Roux and Topisirovic 2012). Several sources of environmental or starvation stressors will trigger a near-global decrease in translation, usually via post-translational modification of a translation initiation factor (Liu and Qian 2014). During these stresses, nontranslating mRNAs may be stored in cytoplasmic RNA storage granules, such as processing (P) bodies or stress granules. P-bodies, which accumulate translation repression and mRNA decay factors (Buchan 2014; Luo et al. 2018), and stress granules, which contain translation initiation factors. Both types of cytoplasmic granules are highly dynamic, with mRNAs thought to move between granules and back into the translating pool (Kedersha and Anderson 2009; Protter and Parker 2016). To understand how translation is regulated, we must understand how mRNAs move between storage and translation.

The essential RNA-dependent helicase Ded1/DDX3 promotes translation and stress granule formation and disassembly. In *Saccharomyces cerevisiae*, Ded1p promotes translation in vivo (Chuang et al. 1997; de la Cruz et al. 1997; Beckham et al. 2008; Lee et al. 2008; Hilliker et al. 2011) and is particularly important for around 1,000 mRNAs, which tend to have structured 5′ untranslated regions (UTRs; Abaeva et al. 2011; Sen et al. 2015; Sen et al. 2019). In purified translation initiation systems, Ded1p promotes stimulation of the 48S preinitiation complex, especially on those highly structured, Ded1-dependent mRNAs (Gupta et al. 2018; Zhou et al. 2024). Ded1p interacts directly with eIF4G (Hilliker et al. 2011), eIF4A (Gao et al. 2016; Gulay et al. 2020), eIF4E (Gulay et al. 2020), and the m7G cap (Senissar et al. 2014). eIF4A and eIF4G modulate Ded1p function (Abaeva et al. 2011; Gao et al. 2016; Gulay et al. 2020) and both Ded1p and DDX3 cooperate with eIF4A on some mRNAs (Soto-Rifo et al. 2012; Sen et al. 2015; Gao et al. 2016). eIF4A, eIF4G, and Ded1 enzymes together provide more helicase activity than eIF4A or Ded1 alone, with Ded1p providing the bulk of the helicase activity in vitro (Gao et al. 2016), consistent with the role of Ded1 in promoting translation of highly structured mRNAs. Taken together, these data suggest that Ded1 works with eIF4F to help with 48S preinitiation complex formation and scanning to the start codon. Ded1p and its mammalian ortholog, DDX3, are highly conserved, as the human and mouse versions can complement a yeast null (Chuang et al. 1997; Mamiya and Worman 1999; Senissar et al. 2014). Direct interactions between the human homolog DDX3 and all of these factors, except eIF4A, have been shown (Shih et al. 2008; Soto-Rifo et al. 2012; Soto-Rifo et al. 2013), demonstrating that the interactions with eIF4F are conserved.

Ded1/DDX3 can also accumulate in various types of cytoplasmic RNA granules, including stress granules (Kanai et al. 2004; Johnstone et al. 2005; Goulet et al. 2008; Lai et al. 2008; Hilliker et al. 2011). Portions of Ded1p's N and C termini promote stress granule formation (Hilliker et al. 2011; Aryanpur et al. 2022) while the ATPase domain promotes stress granule disassembly (Hilliker et al. 2011). Ded1/DDX3 is involved in RNA storage in several contexts where translation regulation is critical. For example, Ded1p orthologs accumulate in neuronal RNA granules (Kanai et al. 2004; Johnstone et al. 2005) and in germ cell granules that are important for development (Salinas et al. 2007). As Ded1 promotes both mRNA translation initiation and accumulation of mRNA-containing stress granules, it might facilitate the transition between translation and storage of mRNAs during stress. Consistent with that model, condensation of Ded1 during heat stress causes formation of stress granules that preferentially contain highly structured housekeeping mRNAs, but not more simply structured stress-dependent mRNAs, suggesting that Ded1 condensation helps reconfigure translation during heat stress (Iserman et al. 2020).

Overexpression of *DED1* in *S. cerevisiae* confers a strong growth defect that correlates with a global drop in translation and increased formation of stress granules (Beckham et al. 2008; Hilliker et al. 2011). Based on our previous characterization of this allele, we propose that overexpression of *DED1* stalls mRNAs with eIF4F and Pab1p in a nontranslating state in stress granules. However, Ded1p's subsequent ATP-dependent role in promoting translation cannot sufficiently disassemble these granules under these conditions (Hilliker et al. 2011), perhaps because some regulator is now limiting compared to the excess Ded1p. Thus, we screened for overexpression suppressors to compensate for the growth defect conferred by overexpression of *DED1.* We hypothesize that such a screen could identify factors that either inhibit Ded1p's first role in interacting with eIF4F or cooperate with Ded1p's subsequent, ATP-dependent role.

In screening for overexpression suppressors, we found a genetic interaction between *DED1* and *HAT1*, a lysine acetyltransferase (KAT) that is known to acetylate free histone H4. We also provide data that *HAT1* can antagonize P-body formation during short-term stresses. During the stationary phase, *HAT1* antagonizes both stress granule formation and survival during the stationary phase. These results suggest an unanticipated functional connection between lysine acetylation and mRNA granule formation modulated by Ded1p. However, we do not find evidence to support a direct relationship, as we do not detect acetylation of Ded1p. Consistent with an indirect connection, we also identified several other acetyltransferases and deacetylases that genetically interact with *DED1* and therefore may influence mRNA regulation.

## Materials and methods

### Plasmid construction

To make a constitutive high copy plasmid with *HAT1* only, *HAT1* ORF (including −520 nucleotides upstream and +499 nucleotides downstream) was amplified from yeast strain BY4741 (Supplementary Table 1 in Supplementary Materials) genomic DNA with primers oAKH183 and oAKH184 to make pAKH257 (*URA3*-marked; Supplementary Tables 2 and 3 in Supplementary Materials). Primers used contained a 40-nucleotide overhang complementary to the ends of the insertion site in the vector backbone. The vector (YEP24) was linearized by digestion with BamHI and XmaI and purified by agarose gel. Half of the PCR reaction of *HAT1* and 150 ng of the linearized vector were transformed into yeast (BY4741) and plated on selective media. Plasmids were isolated from yeast (Zymo Miniprep II, #D2004), amplified in *Escherichia coli*, and confirmed by sequencing. To make a *HIS3*-marked *HAT1* overexpression plasmid (pAKH721), *HAT1* was sub-cloned from pAKH257 into pRS423 by digestion with Xma1 and Sal1. The correct ligation was verified by sequencing.

*FLAG*-*DED1* (pAKH319) or *DED1-FLAG* (pAKH320) contain the endogenous *DED1* gene with an N-terminal or C-terminal FLAG tag, respectively, inserted by QuikChange mutagenesis (Agilent) into plasmid pAKH32. *HAT1* putative catalytic mutants (pAKH290-292) were made by QuikChange. Mutagenesis was confirmed by sequencing.

To make myc-tagged *HAT1* (pAKH834), a myc-tag was incorporated at the C-terminus of wild-type *HAT1* (pAKH257) via SLIM mutagenesis using primers oAKH679-682.

### Yeast strain construction

To make *HAT1-GFP* (yAKH290; Supplementary Table 1 in Supplementary Materials), GFP-KanMx was amplified from pFA6a-GFP(S65T)-KanMX6 (Longtine et al. 1998) with primers oAKH299 and oAKH300, which add 40 nucleotides of homology to the end of the *HAT1* ORF. The PCR product was transformed into the yRP840 yeast and plated on rich media to allow recombination and induction of KanMX6. The next day, cells were replica-plated to YPDA plates containing 0.6 mg/ml G418. Recombinants were verified by G418 resistance; both ends of the GFP insertion were verified by PCR and sequencing from genomic DNA.

### Overexpression suppressor screen

Plasmids containing either an empty vector (pRS423) or a galactose-inducible *DED1* overexpression construct (pAKH201) were transformed into BY4741 yeast. The strain with pAKH201 was transformed with a yeast genomic tiling library of overexpression vectors (Carlson and Botstein 1982; GE Healthcare #YSC5103). The empty vector for this library (YEP24) was transformed into yeast with either pRS423 or pAKH201 to create wild-type (endogenous expression; E) or *DED1* overexpression (OE) controls, respectively. Colonies were replica-plated onto selective media with 2% glucose or with 2% galactose, the latter of which induces *DED1* overexpression. Colonies containing the genomic library that grew larger than the cells with *DED1* overexpression alone were selected and streaked again on selective media with 2% galactose to verify the suppression phenotype.

The suppressor library plasmids were isolated from yeast (Zymo Yeast Miniprep II, #D2004), amplified in bacteria, retransformed into BY4741 yeast with a new copy of pAKH201, and retested for suppression on selective media with 2% galactose. Putative suppressors were identified by sequencing the genomic insert in the plasmid. One putative suppressor from the screen included *HAT1* within a 5.5-kb genomic insert (pAKH245; chromosome XVI, 553662-559121). To test whether *HAT1* was sufficient for suppression, a high-copy plasmid containing the *HAT1* ORF and roughly 500 nucleotides up and downstream was made via recombination (pAKH257), as described above.

### Testing yeast growth phenotypes

Plasmids were transformed into the appropriate yeast strains following the procedure described (Gietz and Schiestl 2007) and plated on the selective media containing 2% glucose.

To test growth with overexpression of *DED1*, cells were grown in patches on solid selective media containing 2% glucose overnight at 30 °C and then re-suspended in selective media containing 2% sucrose. These cultures were normalized to the same optical density (OD_600_ 0.4) and serially diluted before plating on selective media containing either 2% glucose or 2% galactose. The cells were not grown in liquid culture, as that tends to aggravate the accumulation of false positives due to mutations in the galactose-inducible promoter driving *DED1*. All KAT and KDAC deletion strains were from the yeast knockout Mat a collection (Winzeler et al. 1999).

To test the growth of P-body and stress granule deletion strains with galactose-induced overexpression of *HAT1*, cells were grown overnight in liquid selective media with 2% glucose at 30 °C with shaking. Overnight cultures were back-diluted in selective media with 2% sucrose and at 30 °C with shaking. To monitor growth on solid media, mid-logarithmic phase cultures were normalized and plated as described above on selective media with either 2% glucose or galactose. To monitor growth in liquid media, overnight cultures were back-diluted into selective media with either 2% glucose or galactose after cells were washed in that same media. Cell growth was monitored over 18 h at 600 nm in a plate reader.

To test survival from the stationary phase, yeast strains were grown overnight at 30 °C with shaking in selective media with 2% glucose. The next morning, cells were back-diluted in selective media with 2% glucose and 100 μg/μl ampicillin. At mid-log phase, a portion of each culture was concentrated to an equivalent of OD_600_ of 1.5 and plated in 5-fold serial dilutions on selective media. The cultures continued to grow at 30 °C with shaking for 9 d. Each day, the cells were visualized by microscopy for cytoplasmic granules (see below) and plated in normalized serial dilutions, as described. All plates were incubated at 30 °C.

### Western blot analysis of Ded1p levels

BY4741 cells were transformed with a plasmid with *DED1* regulated by a galactose-inducible promoter (overexpression or OE; pAKH201) or the corresponding plasmid backbone with no *DED1* (endogenous levels or E; pRS423). These cells were also transformed with a plasmid that constitutively overexpresses *HAT1* (OE; pAKH257) or the equivalent plasmid with no *HAT1* (E; YEP24). Transformants were grown overnight in selective media with 2% glucose. Cultures were back-diluted in the morning in selective media with 2% sucrose and grown to mid-log phase. Cells were washed in selective media with 2% galactose and then re-suspended in the same media type, and then grown at 30 °C with shaking for 14 h to induce *DED1* expression. Cells were disrupted by bead bashing and boiling in 125 mM Tris–HCl, pH 6.8, 2% SDS, 1 M urea. Protein lysate concentration was determined by Bradford assay. Equal concentrations of each lysate were loaded on a 10% TGX Stain-Free PAGE gel (BioRad), and equal protein levels were verified by Stain-Free imaging. Blots were probed with anti-Ded1p antibody as previously described (Hilliker et al. 2011). See Supplemental Methods for details on tracking Pab1 and Edc3 levels by western blot (Supplementary Materials).

### Immunoprecipitation of Ded1p

BY4741 yeast cells were transformed with a plasmid containing C-terminally FLAG-tagged *DED1* (pAKH320) or an empty vector (pRS413). To test the effect of constitutive overexpression of *HAT1*, a high-copy plasmid with *HAT1* (pAKH257) or an empty vector (YEP24) was also transformed. Strains were grown to mid-log phase in selective media with 2% glucose. A 50 ml of mid-log culture was pelleted, washed, and re-suspended in 400 μl of lysis buffer (50 mM Tris–HCl, pH 7.5, 150 mM NaCl, 2 mM MgCl_2_, 0.2% NP-40, 1 mM β-mercaptoethanol, Pierce EDTA-free protease inhibitors). Cells were disrupted by bead beating with an equal volume of acid-washed glass beads. Supernatant was clarified by spinning at 20,000*×g* for 10 min at 4 °C. Each sample was incubated with 40 μl of 50% slurry of anti-FLAG agarose resin (Sigma) for 2 h at 4 °C. Beads were washed 3 times in wash buffer (50 mM Tris–HCl, pH 7.5, 500 mM NaCl, 2 mM MgCl_2_, Pierce EDTA-free protease inhibitors). FLAG-Ded1p was eluted by either incubation with 50 μl of wash buffer with 0.2 mg/ml 3 × FLAG peptide or by boiling in Laemmli Sample Buffer. The sample was loaded on 10% TGX Stain-Free PAGE gels (BioRad). Blots were probed with anti-Ded1p antibody as previously described (Hilliker et al. 2011) and 2 different pan antiacetyl lysine antibodies (first with Cell Signaling Technology Cat# 9441, RRID:AB_331805 and then with Sigma–Aldrich Cat# MABE1020, RRID:AB_3719374). Recombinant Ded1p and acetylated BSA were loaded as controls for the antibodies.

### In vitro activity of yeast Hat1

To immobilize the Hat1 protein from yeast, a haploid yeast strain with a knockout of *HAT1* (yAKH201) was transformed with plasmids containing either no additional gene (YEP24) or myc-tagged wild-type *HAT1* (pAKH834), plated on selective media, and grown at 30 °C. One liter of each yeast culture was grown to mid-log phase in selective media at 30 °C. The culture was chilled on ice for 10 min and then cells were pelleted. Pellets were washed in 2 times volume per gram of cell pellet in cold water and then washed in 1.3 × volume with extraction buffer (100 mM Hepes-KOH pH 8.2, 245 mM KCl, 5 mM EGTA, 1 mM EDTA, 2.5 mM DTT). The pellet was resuspended in 1.3 × volume (per gram of cells) of extraction buffer plus 1 × protease inhibitor cocktail (Pierce, A32955). Cell slurry was dripped into liquid nitrogen and the frozen yeast pellets were disrupted by grinding with a mortar and pestle under liquid nitrogen. After centrifugation to remove cell debris, myc-tagged Hat1p was purified using 25 µl of anti-c-Myc agarose resin (Thermo, 20168). Resin was washed 3 times with 20 times the resin volume of Tris-buffered saline plus 0.05% Tween-20 (TBS-T). Resin was resuspended in 6 times the resin volume of TBS-T.

The activity of the purified yeast Hat1 proteins was tested under conditions similar to those previously published (Mersfelder and Parthun 2008). Fifty microliter reactions included 15 µl of resin with immobilized myc-tagged Hat1p from the yeast lysate (or resin incubated in lysate from cells without Hat1), which represents 10% of the immobilized protein. The resin was incubated with 1 × DN(50) buffer (25 mM Tris–HCl pH 7.9, 0.1 mM EDTA, 10% glycerol, 50 mM NaCl), and 2.5 mM [^3^H] acetyl coenzyme A (200 mCi/mmol; BioTrend, #ART-0213A-1). Either 0.2 mg/ml human histone H4 (Sigma H2667) or 0.2 mg/ml of recombinant Ded1p (Hilliker et al. 2011) was added as substrate, and reactions were incubated for 60 min at 37 °C. Supernatants from reactions were passed over P81 ion exchange cellulose chromatography paper (Reaction Biology Corp., #IEP-01), which were then washed 3 times with 5 ml of 50 mM NaHCO_3_ (pH 9.0). Filters were air-dried and then tritium levels were quantified by liquid scintillation counting.

### Microscopy of P-bodies and stress granules

Hat1p-GFP strains were transformed with either a P-body marker (Edc3p-mCh; pAKH96) or stress granule marker (Pub1p-mCh; pAKH94). For all other experiments, yeast strains were transformed with a plasmid co-expressing *EDC3-mCh* and *PAB1-GFP* as P-body and SG markers, respectively (pAKH51). Yeast cultures were grown overnight in selective media with 2% glucose, diluted to an OD_600_ of 0.1, and grown to mid-log phase before applying short-term stresses, as described below.

For sodium azide stress, cells were treated with 0.5% sodium azide. An equivalent volume of water was added to mock-stressed cells. All samples were incubated at 30 °C for 30 min with shaking and visualized as described below. For glucose deprivation stress, cells were pelleted at a low speed and washed once with selective media lacking glucose. Cells were re-suspended in the initial volume of selective media lacking glucose. Samples were incubated at 30 °C for 15 min and visualized as described below. For diauxic shift or stationary phase stress, overnight cultures, grown in selective media with 2% glucose, were back-diluted in selective media with 2% glucose and 100 μg/μl ampicillin and grown to mid-log phase at 30 °C with shaking. Cells were grown for 9 d at 30 °C with shaking with ampicillin added again at day 5. Cells were visualized daily. Day 1 or nonstress conditions represent cells in mid-logarithmic growth. Cells imaged on day 2 are in diauxic shift. By day 5 or 6, yeast enters the stationary phase.

For each stress, at least 3 independent experiments were performed, each with at least 100 cells imaged in each experiment. Z-series images were taken at 100 × magnification. A range of 4.5 µm was visualized in the Z-series with a step size of 0.3 µm. Z-series images were collapsed and normalized using FIJI software (RRID:SCR_002285). The images were captured on an Olympus BX61/DG4 microscope with a Hamamatsu ORCA-ER camera (#C4742-80) or on an Olympus IX83 microscope with a Hamamatsu ORCA-Flash4.0 camera (#C11440). Both the average number of granules per cell and the percentage of cells with visible granules were measured in FIJI (as in Buchan et al. 2010). All *P*-values were calculated from unpaired two-tailed *t*-tests.

## Results and discussion

### Genetic interaction between *HAT1* and *DED1*

**Fig. 1.**
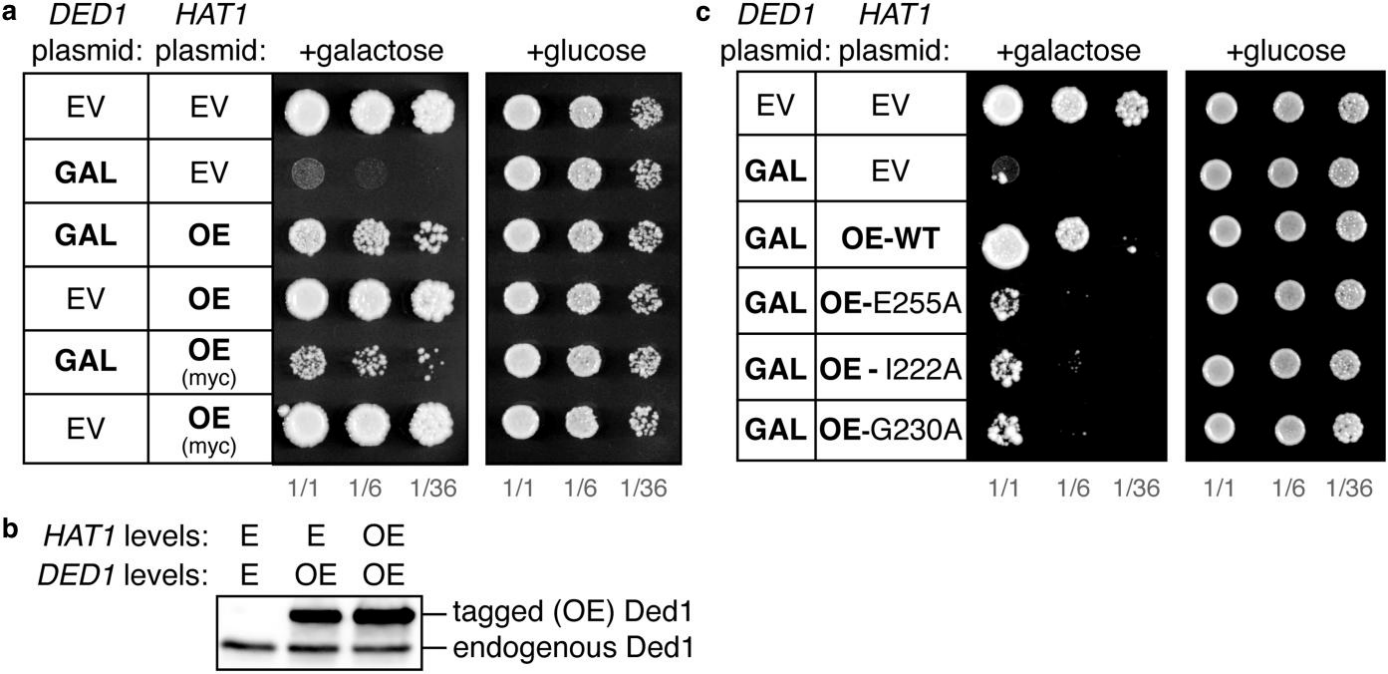
Overexpression of *HAT1* suppresses the growth defect conferred by overexpression of *DED1*. a) Yeast strain BY4741 was transformed with a plasmid containing no additional DED1 (EV, or empty vector pRS423), to assess endogenous expression of *DED1*, or a plasmid containing galactose-inducible overexpression of *DED1* (GAL, or pAKH201, which causes overexpression only on galactose-containing media). These strains were co-transformed with either another empty vector (EV, or empty vector YEP24), to test endogenous expression of *HAT1,* or a plasmid that constitutively overexpresses *HAT1* (OE, pAKH257, which overexpresses *HAT1* on any media). The strains were pin replicated at the same concentration by serial dilution on selective media containing 2% glucose or galactose. Cells were grown for 8 (left) or 3 d (right). b) Equal amounts of whole cell extracts were analyzed via western blot with an anti-Ded1p antibody to assess levels of Ded1p in each strain after a 14-hour induction of *DED1* in galactose-containing media. Ded1p from the overexpression construct was tagged, allowing visualization of endogenous (E) and overexpressed, tagged Ded1p (OE). c) Alterations to amino acids in *HAT1* that are predicted to decrease catalytic activity (pAKH290-292) were each transformed into a *hat1* null. wild-type or mutant *HAT1* alleles were tested for their ability to suppress the defect of cells overexpressing *DED1.* Cells are plated as in (a), except that cells grew for 7 d (left) or 2 d (right), and cells were deposited by a multichannel pipette.

To identify regulators of Ded1p, we selected for overexpression suppressors of the strong growth defect conferred by excess *DED1*. Yeast expressing a high-copy plasmid with galactose-inducible *DED1* has a severe growth defect on galactose-containing media due to *DED1* overexpression (Fig. 1a; Hilliker et al. 2011). We introduced a *S. cerevisiae* genomic overexpression library into this yeast strain and screened for colonies that suppressed the strong growth defect caused by excess Ded1p. In our screen, we identified a genomic fragment (chromosome XVI 553662-559121) that suppressed the growth defect of *DED1* (Supplementary Fig. 1 in Supplementary Materials). This genomic fragment contained full-length ORFs *CIT3*, a mitochondrial citrate synthase (Jia et al. 1997), and *HAT1*, a histone acetyltransferase. Hat1p acetylates free histone H4 and H2A and has been implicated in DNA repair, DNA replication, histone turnover (Qin and Parthun 2002; Suter et al. 2007; Verzijlbergen et al. 2011), telomeric silencing (Kelly et al. 2000; Mersfelder and Parthun 2008), and telomeric position effect (Power et al. 2011), all of which involve either the placement of new chromatin or heterochromatin maintenance (Ortega et al. 2023).

Despite its well-studied role in chromatin formation, we hypothesized that *HAT1* was causing the suppression, as it does show some cytoplasmic localization (Kleff et al. 1995; Parthun et al. 1996; Ruiz-Garcia et al. 1998; Verreault et al. 1998; Poveda and Sendra 2008) and co-purifies with proteins involved in mRNA regulation, including subunits of translation initiation factor eIF3, ribosomal protein RPL37A, and translation repression factor Stm1 (Gavin et al. 2002; Krogan et al. 2006).

To determine if *HAT1* is the relevant gene on the suppressor plasmid, we cloned the *HAT1* gene into a high-copy vector and observed that constitutive *HAT1* overexpression is sufficient to confer suppression (Fig. 1a). Since the original phenotype in this screen depends on overexpression of *DED1* and some acetyltransferases can affect transcription, we tested whether overexpression of *HAT1* affects Ded1p protein levels. We can differentiate between Ded1p expressed from the chromosome and from the plasmid, as the plasmid copy of *DED1* has a large C-terminal tag. As observed by western blot, neither the levels of chromosomally expressed Ded1p nor overexpressed Ded1p were altered by overexpression of *HAT1* (Fig. 1b), suggesting that *HAT1* overexpression does not suppress by lowering Ded1 protein levels. This is consistent with Hat1's known role in only acetylating free histone H4, not H4 packaged into nucleosomes and chromatin; yeast lacking the *HAT1* gene have no defect in transcription, as shown by microarray or RNA polymerase occupancy (Parthun et al. 1996).

To test whether *HAT1* suppression depends on the catalytic activity of Hat1p, we made mutations in sites known or hypothesized to affect Hat1p catalytic function. The glutamic acid at position 255, when mutated to glutamine, causes a 10-fold decrease in acetylation of histone H4 in vitro, demonstrating that this amino acid promotes Hat1p catalytic activity (Mersfelder and Parthun 2008). The crystal structure of Hat1p with acetyl CoA shows that isoleucine 222 and glycine 230 directly interact with the acetyl CoA substrate (Dutnall et al. 1998). Mutations in homologous positions reduce catalytic activity in other lysine acetyltransferases (Tercero et al. 1992; Coleman et al. 1996; Lu et al. 1996; Kuo et al. 1998; Smith et al. 1998). We individually mutated these positions to alanine and tested whether overexpression of these mutants could still suppress the growth defect from overexpression of *DED1*. While the *hat1* mutants showed weak suppression of the *DED1* overexpression defect, they did not suppress nearly as well as wild-type *HAT1* (Fig. 1c), suggesting that Hat1p catalytic activity may be important for the genetic interaction with *DED1.* A caveat to this work is that we have not developed assays to test whether the overexpression of *HAT1* increases acetylation of known in vivo targets, nor have we tracked Hat1 protein levels in vivo when overexpressing wild-type vs mutant *HAT1*.

### The genetic connection between Ded1p and Hat1p may not be due to direct acetylation

It is increasingly clear that many KATs, including Hat1p, can have nonhistone targets, including some RNA-dependent ATPases (Poziello et al. 2021). In mouse germ chromatoid bodies, Hat1p acetylates the RNA helicase MVH (Mouse Vasa Homolog or DDX4), which decreases its RNA binding ability and its ability to repress translation (Nagamori et al. 2011). Other DEAD-box ATPases are known to be acetylated by different KATs. Acetylation of human helicases p68 and p72 promotes their function in transcription (Mooney et al. 2010). RNA helicase A (DHX9) in humans has been implicated in translation (Hartman et al. 2006) and is acetylated (Chuang et al. 2010), but the function of its acetylation is unknown.

**Fig. 2.**
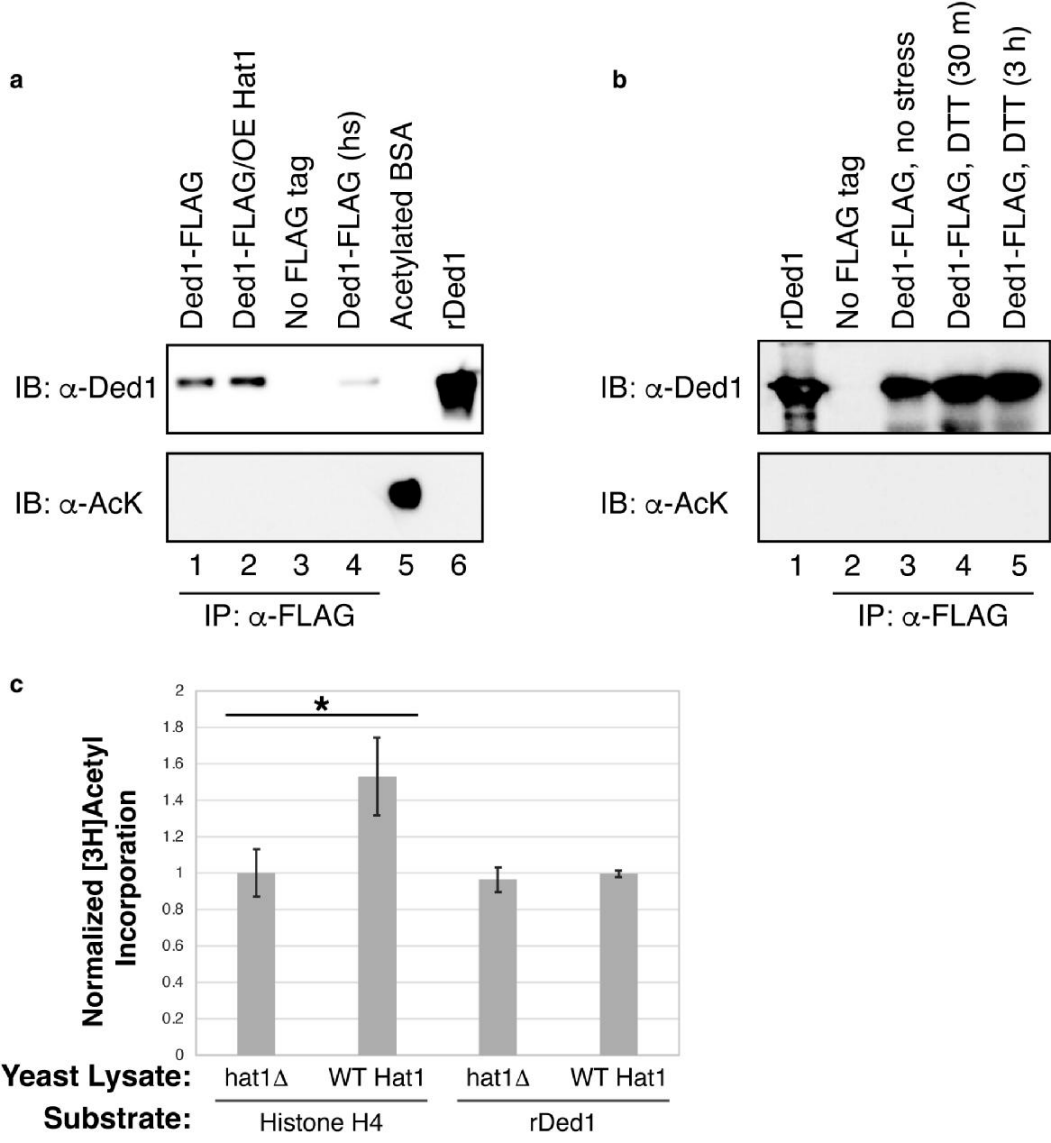
Ded1p is not detectably acetylated in vitro. a and b) Ded1p tagged with FLAG (pAKH320) was immunoprecipitated from BY4741 yeast either expressing endogenous (YEP24) or constitutively overexpressed levels of *HAT1* (pAKH257). The samples were assessed for the presence of acetylated proteins using an antiacetyl lysine pan antibody. The levels of immunoprecipitated Ded1p were visualized by an anti-Ded1p antibody. Purified acetylated BSA (50 ng) and recombinant Ded1p (800 ng) were used as controls for the antibodies. Yeast cells were grown to mid-log phase in selective media without stress unless indicated. Stresses included 30 min of high heat stress at 46 °C (hs) or DTT for 30 min or 3 h. c) Yeast lysate containing no *HAT1* (yAKH201 with YEP24) or containing myc-tagged Hat1p (yAKH201 + pAKH834) was incubated with anti-c-Myc agarose resin to immobilize Hat1p, if present. Resin was incubated with 2.5 mM [^3^H] acetyl coenzyme A and 0.2 mg/ml of either human histone H4 or recombinant Ded1p for 60 min at 37 °C. Protein was captured on chromatography paper, and tritium levels were quantified by liquid scintillation counting. Bars represent the average of triplicates; error bars represent the SD. *P*-values less than 0.05 as calculated by an unpaired two-tailed *t*-test are marked with an asterisk.

To test whether Ded1p is acetylated in yeast, we immunopurified FLAG-tagged Ded1p from wild-type yeast or yeast overexpressing *HAT1* (Fig. 2a), reasoning that overexpression of *HAT1* would increase any acetylation signal. This FLAG-tagged version of Ded1p can complement a *ded1* null (Supplementary Fig. 2 in Supplementary Materials), showing that it retains the essential function of Ded1p. We tested for the efficiency of immunoprecipitation by probing with an anti-Ded1p antibody (Fig. 2a). We tested whether the purified Ded1p was acetylated by probing with 1 of 2 different pan antiacetyl-lysine (α-AcK) antibodies, one of which was used to identify the acetylation of MVH (Nagamori et al. 2011). While we were able to immunoprecipitate Ded1p-FLAG (Fig. 2, a and b, top), we did not detect any Ded1p acetylation signal by western blot in cells with endogenous or overexpressed *HAT1* (Fig. 2, a and b, lanes 1 to 2, bottom). We used acetylated BSA as a control to ensure that the antibody conditions were appropriate.

Both Hat1p and Ded1p have connections to stress responses, so we tested whether a variety of stresses would increase acetylation to detectable levels. Hat1p helps protect *Candida albicans* against oxidative stress (Tscherner et al. 2012). Ded1p promotes translation and accumulates in stress granules (Hilliker et al. 2011; Aryanpur et al. 2022), where nontranslating mRNPs accumulate during many cellular stresses (Buchan 2014). We tested whether Ded1p-FLAG was acetylated under the following cellular stresses: oxidative stress (hydrogen peroxide or menadione treatment), sodium azide, ER stress (DTT or cadmium treatment), glucose starvation, amino acid starvation, diauxic shift into the stationary phase, and high heat stress. However, we did not observe detectable acetylation under any of these conditions (2 stresses are shown in Fig. 2b; the rest are not shown). Although we used a similar method and antibodies to those used to detect acetylation of MVH, we found no evidence that Ded1p is acetylated. However, it is possible that Ded1p is acetylated under different conditions or that it is acetylated with an efficiency too low to be detected by these antibodies.

We also tested whether Hat1p could acetylate Ded1 in vitro. We immobilized myc-tagged Hat1p complexes under native conditions from yeast and incubated the immobilized Hat1p with tritium-labeled acetyl CoA and either human histone H4 peptide or recombinant Ded1p. Histone H4 peptide was detectably acetylated by immobilized Hat1p, compared to resin incubated in yeast lysate lacking Hat1p. However, we could not detect any acetylation of Ded1p by immobilized Hat1p (Fig. 1c). If Ded1p is acetylated by Hat1p, we could not detect it in either our in vivo or in vitro assays.

This lack of acetylation is consistent with the literature, which shows convincing evidence that the human ortholog of Ded1p, DDX3X, is acetylated and functionally significant, but lacks evidence that this acetylation is conserved in yeast. Several proteomic analyses in mammalian cells show acetylation of DDX3X, although different positions (K50, K55, K64, K66, K81, and K118) are identified in each screen (Choudhary et al. 2009; Chen et al. 2012; Lundby et al. 2012; Schölz et al. 2015; Saito et al. 2019). Deacetylation of DDX3X, catalyzed by HDAC6, promotes granule formation (Saito et al. 2019), suggesting a functional role for DDX3X acetylation. The most reproducible and robust modification is acetylation of DDX3X-K118 by acetyltransferases CBP or p300, which antagonizes both DDX3X condensate formation and stress granule maturation (Saito et al. 2019). Of the lysines that have some level of acetylation in DDX3X, none of these positions are conserved in Ded1p (based on protein alignments from Garbelli et al. 2011).

In contrast, the evidence for yeast Ded acetylation is sparse. In a proteomic immunoprecipitation and mass spectrometry screen for proteins with acetylated lysines, Ded1p positions K92 and K164 were identified as acetylated in 1 of 4 replicates from mid-log growth, while K246 was found to be acetylated in 1 of 2 replicates from cells lacking *RPD3* (Henriksen et al. 2012). Another proteomic screen calculated the stoichiometry of acetylation and found very low levels of acetylation for cytoplasmic proteins, including Ded1p-K246 (Weinert et al. 2014). Nonenzymatic acetylation can occur at physiological levels of acetyl-CoA (Weinert et al. 2014), so it is unclear whether this acetylation stems from an acetyltransferase. Only K92 is conserved in DDX3X (Garbelli et al. 2011), but currently, there is no evidence that this position is acetylated in DDX3.

Given the lack of support that Hat1p directly acetylates Ded1p, *HAT1* may suppress *DED1* by acetylating other proteins that affect Ded1p's function in translation or stress granule accumulation. A proteomic screen of acetylation in yeast proteins was enriched in factors related to translation, translation initiation, and translation regulation. Several translation initiation factors that have either physical or genetic connections to Ded1p are acetylated, including eIF4A, eIF4E, and eIF3 subunits in yeast (Henriksen et al. 2012) and eIF4G in HeLa cells (Chuang et al. 2010). Future work could explore whether Hat1p could acetylate these factors and thereby influence Ded1p function.

### Overexpression of *HAT1* exacerbates the growth defect on solid media of an *lsm4* truncation

**Fig. 3.**
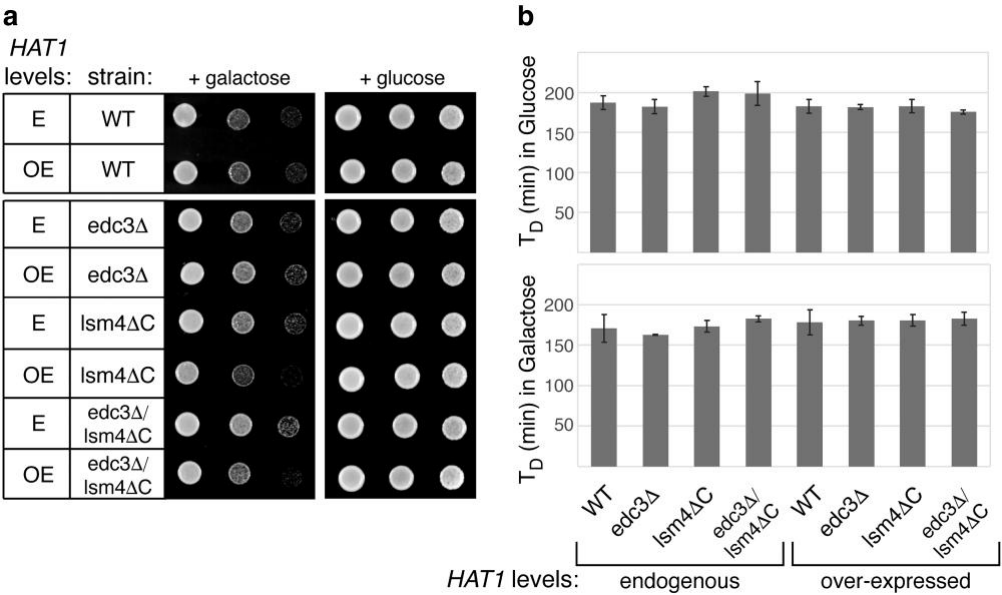
Overexpression of *HAT1* has a slight synthetic effect with *lsm4ΔC*, but not *edc3Δ*, when grown on solid media. Yeast cells lacking either *EDC3,* the C-terminal domain of *LSM4,* or both (yAKH210, yRP2337-2338; Supplementary Table 1 in Supplementary Materials) were transformed with either an empty vector to measure growth with the endogenous levels of Hat1p (E; pAKH306) or with galactose-inducible *HAT1* to measure growth in the presence of overexpression of *HAT1* (OE; pAKH258). a) Strains were plated at the same concentration on solid minimal media with 2% galactose, where *HAT1* overexpression is induced, or 2% glucose and grown for 2 d at 30 °C. b) Doubling time (*T*_D_) was calculated from growth in liquid minimal media with either 2% glucose (top) or 2% galactose (bottom) over 18 h at 30 °C. The bars represent the average of 3 replicates and the error bars show the SD. There was no significant difference in the doubling time of the mutant strains compared to the wild type control in either medium (unpaired two-tailed *t*-test).

As Ded1p influences P-body and stress granule formation (Hilliker et al. 2011), we hypothesized that the genetic interaction between *HAT1* and *DED1* may manifest via an effect of Hat1p on P-body or stress granules. We tested whether the overexpression of *HAT1* could affect the growth of yeast strains defective in stress granule or P-body formation. Pub1p and Pbp1p are stress granule components that are important for stress granule formation, as yeast lacking *PUB1* or *PBP1* are defective in stress granule formation (Buchan et al. 2008). However, overexpression of *HAT1* had no effect on the growth of *pub1Δ* or *pbp1Δ* yeast (Supplementary Fig. 3 in Supplementary Materials). Edc3p and Lsm4p are P-body components that are important for P-body formation. Yeast lacking *EDC3* are defective in P-body formation, while yeast lacking *EDC3* and the C terminus of *lsm4* show a stronger defect in P-body formation (Decker et al. 2007; Reijns et al. 2008). Interestingly, overexpression of *HAT1* did not affect an *edc3* null mutation but did slightly exacerbate the growth on solid media of the *lsm4ΔC* mutation either in the presence or absence of *EDC3* (Fig. 3a). Since these phenotypes do not correlate with the P-body formation defects, they may reflect a relationship between *HAT1* and *LSM4* that is independent of its P-body assembly role but is consistent with some connection between acetylation and mRNA regulation. However, there was no synthetic effect between *lsm4ΔC* and the overexpression of *HAT1* when cells were grown in liquid (Fig. 3b). The difference in phenotype between growth on solid vs in liquid media likely reflects the challenge of growing on solid media, where cells within the patch do not have equal access to water and nutrients. This more stringent growth environment might reveal a defect that is not limiting in liquid culture, where all cells have access to nutrients.

While the C-terminus of Lsm4p promotes P-body formation in both yeast and humans (Kedersha et al. 2005; Decker et al. 2007; Reijns et al. 2008), its C-terminus is highly divergent. In yeast, the C-terminus is (Q/N)-rich, whereas in metazoans, it is (R/G)-rich. The human ortholog of Hat1p has been shown to interact with the C-terminus of human Lsm4p (Arribas-Layton et al. 2016), but we show that overexpression of *HAT1* worsens the growth defect of an *lsm4* C-terminal truncation, suggesting that the relationship between Hat1p and Lsm4p may have changed between these eukaryotes.

### *HAT1* antagonizes granule accumulation during short-term stresses

Cytoplasmic RNA storage granules, including P-bodies and stress granules, are sensitive indicators of nontranslating mRNAs. In nonstressed cells, P-bodies are rare, and stress granules are absent, but both accumulate when translation is inhibited (Kedersha and Anderson 2009; Buchan 2014). We reasoned that an indirect effect of Hat1 on Ded1 might alter granule formation. To evaluate if *HAT1* affects mRNA granules, we tested whether the overexpression or the absence of *HAT1* alters the formation of P-bodies or stress granules under short-term stresses.

**Fig. 4.**
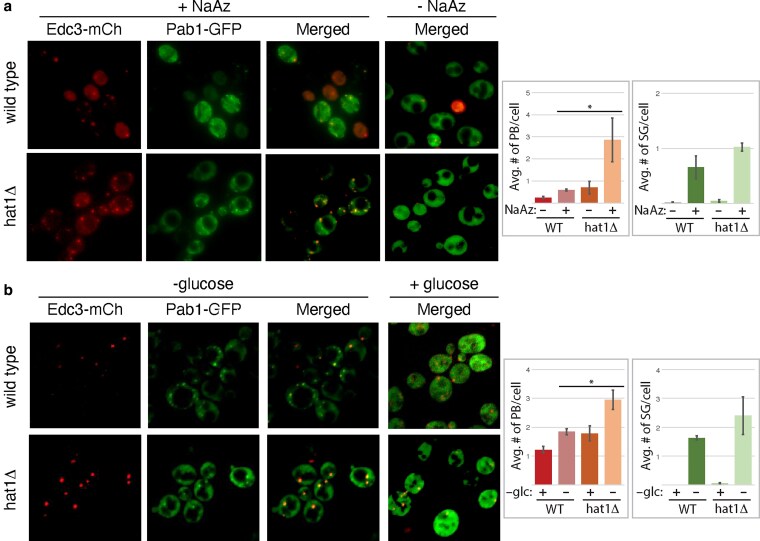
Cells lacking *HAT1* show an increase in P-bodies under short-term stress. a and b) wild-type yeast (BY4741) or yeast with *hat1*Δ (yAKH201) were transformed with a plasmid containing Edc3p-mCh, a P-body marker, and Pab1p-GFP, a SG marker (pAKH51). To induce stress granule formation, wild-type and *hat1Δ* yeast were grown to mid-log phase in selective media with 2% glucose and imaged after either a mock treatment or after a stress treatment. Cells were stressed with a) 0.5% sodium azide for 30 min or b) glucose deprivation for 15 min. The average number of P-bodies (PB) or stress granules (SG) per cell is graphed at right; the percentage of cells with cytoplasmic granules is shown in Supplementary Fig. 4 (in Supplementary Materials). The error bars represent the SD. For all samples shown, an unpaired two-tailed *t*-test evaluating each strain with or without stress generated a *P*-value of less than 0.05. When comparing wild-type (WT) vs mutant strains under stress conditions, *P*-values less than 0.05 are indicated with an asterisk. Pictures show representative samples from at least 3 independent experiments visualizing at least 300 cells.

**Fig. 5.**
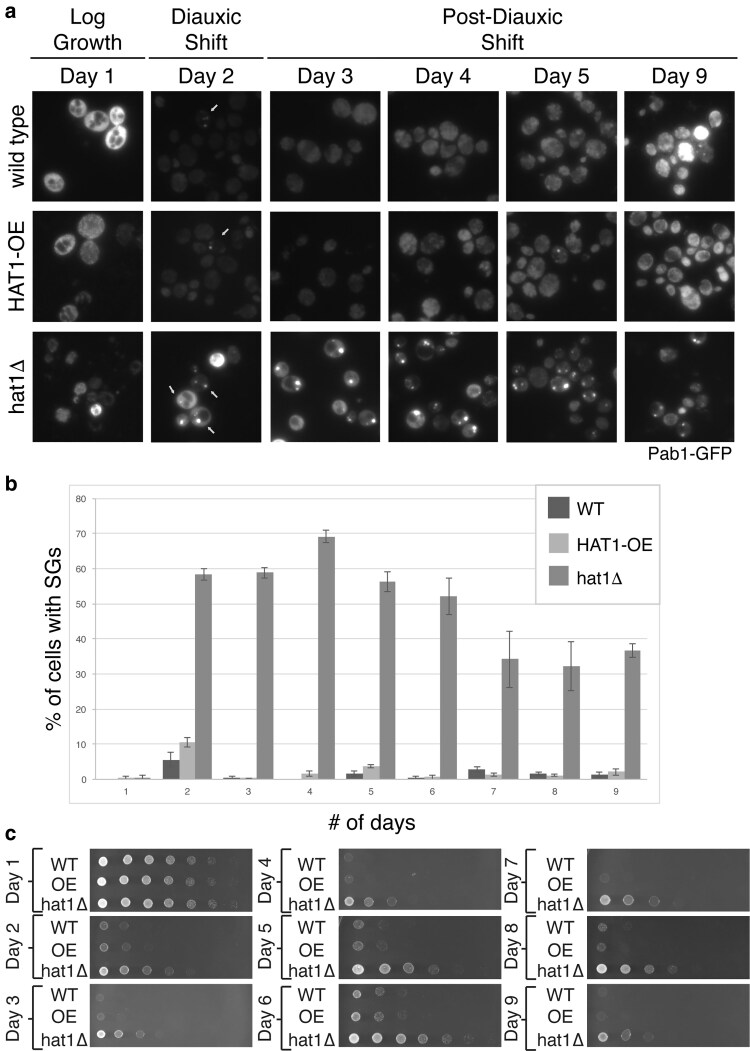
Deletion of *HAT1* causes an increase in stress granule formation and cell survival during the stationary phase. Wild-type yeast (WT; BY4741 with pRS423), yeast overexpressing *HAT1* (OE; BY4741 with pAKH721), or yeast lacking *HAT1* (hat1Δ; yAKH201 with pRS423) were grown in selective media with 2% glucose. On day 1, cells were in logarithmic growth phase. a) Stress granules were visualized daily with Pab1p-GFP (pAKH51). P-bodies were visualized by Edc3p-mCh but did not change between mutant and wild-type (Supplementary Fig. 5 in Supplementary Materials). White arrows indicate cells with stress granules. b) Bar graph shows the average percentage of cells with stress granules from the experiment shown in (a). The error bars represent the SE of the mean. At least 200 cells were assayed for each replicate. A unpaired two-tailed *t*-test between WT and hat1Δ strains on days 2–9 showed a *P*-value < 0.05. c) The same concentration of cells was plated onto selective media daily for 9 d; plated cells were incubated at 30 °C for 1 d (or 2 d as shown in Supplementary Fig. 5b in Supplementary Materials). Images show representative samples from one of at least 3 independent experiments.

We assayed cytoplasmic granule formation in strains deleted for the *HAT1* gene (*hat1Δ*) in the presence of fluorescently tagged granule proteins. As P-body and stress granule composition and assembly requirements vary by stress (Buchan et al. 2011), we tested the effect of granule formation under several stresses: glucose deprivation, sodium azide treatment, and diauxic shift (Figs. 4 and 5). Compared to wild-type yeast, the absence of *HAT1* causes a statistically significant increase in the number of P-bodies per cell during stress with either sodium azide treatment or short-term glucose deprivation (Fig. 4, a and b). The overall percentage of cells that formed P-bodies upon sodium azide stress did not change between wild-type and *hat1Δ* yeast. There was no statistical difference in stress granule formation in yeast lacking or overexpressing *HAT1*, compared to wild-type yeast (Fig. 4, Supplementary Fig. 4a and b). These data suggest that *HAT1* normally antagonizes P-body formation or accumulation during glucose deprivation and sodium azide treatment.

### Deletion of *HAT1* causes increased stress granule formation and cell survival during stationary phase

The metabolic state of the cell greatly influences the activity of KATs. KAT enzymes use acetyl CoenzymeA (CoA) as their substrate, which is in abundance when glucose is available to drive glycolysis. Extended yeast growth in culture eventually exhausts stores of glucose, causing available pools of acetyl CoA to decrease, limiting KAT function. Consistent with this, histone acetylation levels parallel the concentration of cellular acetyl-CoA (Galdieri et al. 2014). Presumably, acetylation of cytoplasmic proteins follows the same pattern.

As the yeast metabolize glucose, it produces ethanol (Diaz-Ruiz et al. 2009). After about a day of growth, the yeast cells undergo diauxic shift, where the yeast begin to metabolize the ethanol and grow more slowly. They continue in this phase, called post-diauxic shift (PDS), for the next 4 or 5 d (Gray et al. 2004) before entering the true stationary growth phase (Werner-Washburne et al. 1993; Herman 2002). During these growth stages, P-body formation correlates with diauxic shift (Teixeira et al. 2005) and persists through the stationary phase (Shah et al. 2013). Stress granules also form during diauxic shift (Brengues and Parker 2007).

The stationary phase includes an extended time in growth arrest under starvation conditions. We tested whether varying *HAT1* levels affected cytoplasmic granule formation or cell survival during diauxic shift and the stationary phase. Samples were taken from the cultures daily over 9 d, analyzed by microscopy, and plated at equivalent concentrations onto selective media to monitor their ability to form colonies. All strains plated from the 1-d cultures, which were in logarithmic growth, grew like wild-type yeast. After 2 d of growth, when cells are undergoing diauxic shift, strains lacking *HAT1* had a distinct growth advantage over wild-type cells and cells overexpressing *HAT1* (Fig. 5c and Supplementary Fig. 5b). The growth advantage conferred by *hat1Δ* was maintained over the 9 d of growth we tested. This growth advantage in the stationary phase is the first easily tractable growth phenotype observed in the *hat1* null mutant, providing a useful genetic tool for future genetic studies on *HAT1* function.

The growth advantage conferred by *hat1Δ* was maintained over 9 d of growth and correlates with the onset of stress granules on day 2. During diauxic shift (day 2), yeast lacking *HAT1* showed a 5-fold increase in the percentage of cells containing stress granules, compared to wild-type cells. These stress granules persisted in the *hat1Δ* cells over the 9 d of the experiment, showing a partial decline over time (Fig. 5, a and b). Pab1 protein levels in *hat1*Δ cells actually decrease over time, even as stress granules persist (Supplementary Fig. 6 in Supplementary Materials). These results suggest that wild-type *HAT1* has a negative effect on stress granule accumulation.

Others have shown that survival during the stationary phase correlates with the ability to form P-bodies (Shah et al. 2013), and we observe that *hat1Δ* yeast forms more P-bodies under short-term stresses (Fig. 4). Therefore, we also tested whether *hat1Δ* alters P-body formation during diauxic shift. Unlike stress granules, P-body formation was consistent during diauxic shift and the stationary phase, even in the absence of *HAT1* (Supplementary Fig. 5, a and b in Supplementary Materials) and a slight drop in Edc3 protein levels in *hat1Δ* cells (Supplementary Fig. 6 in Supplementary Materials). Changes in P-body formation did not correlate with changes in growth in *hat1Δ* cells.

Our data shows the first correlation between stress granule formation and increased survival during the stationary phase and reveals a condition where P-body levels do not increase with stationary phase survival, as seen previously (Shah et al. 2013). While the correlation between sustained stress granule (Fig. 5) or P-body formation and increased survival during the stationary phase is intriguing, it is unclear whether cytoplasmic RNA granules directly contribute to survival in the stationary phase. In mammalian cells, stress granule formation antagonizes apoptosis by sequestering proapoptotic factors (Arimoto et al. 2008). Perhaps the stress granules formed in the absence of *HAT1* sequester similar factors. Further study of the composition of P-bodies and stress granules formed under these conditions may illuminate whether either type of cytoplasmic granule impacts survival in the stationary phase.

Our work contributes to other data suggesting a link between acetylation and cytoplasmic RNA granule formation. The deacetylase HDAC6 promotes mammalian stress granule formation and localizes to stress granules (Kwon et al. 2007). In yeast, deletion of one or more KDACs (*hos3Δ* alone, *sir2Δ/hst1Δ/hst2Δ*, or *sas2Δ/sas3Δ*) decreases stress granule formation under glucose deprivation stress (Rollins et al. 2017; Sivananthan et al. 2023). Acetyltransferase Gcn5p and deacetylases Hst1p and Sir2p antagonize stress granules in nonstressed cells (Buchan et al. 2013), while the acetyltransferase complex NuA4 promotes the formation of stress granules during glucose deprivation specifically. Interestingly, supplementing media with exogenous acetate decreases the formation of glucose deprivation stress granules, suggesting that stress granule formation may be sensitive to acetyl CoA levels (Rollins et al. 2017) under some stresses, in which case, we might expect several KATs and KDACs to impact stress granules by affecting the pool of available acetyl CoA.

**Fig. 6.**
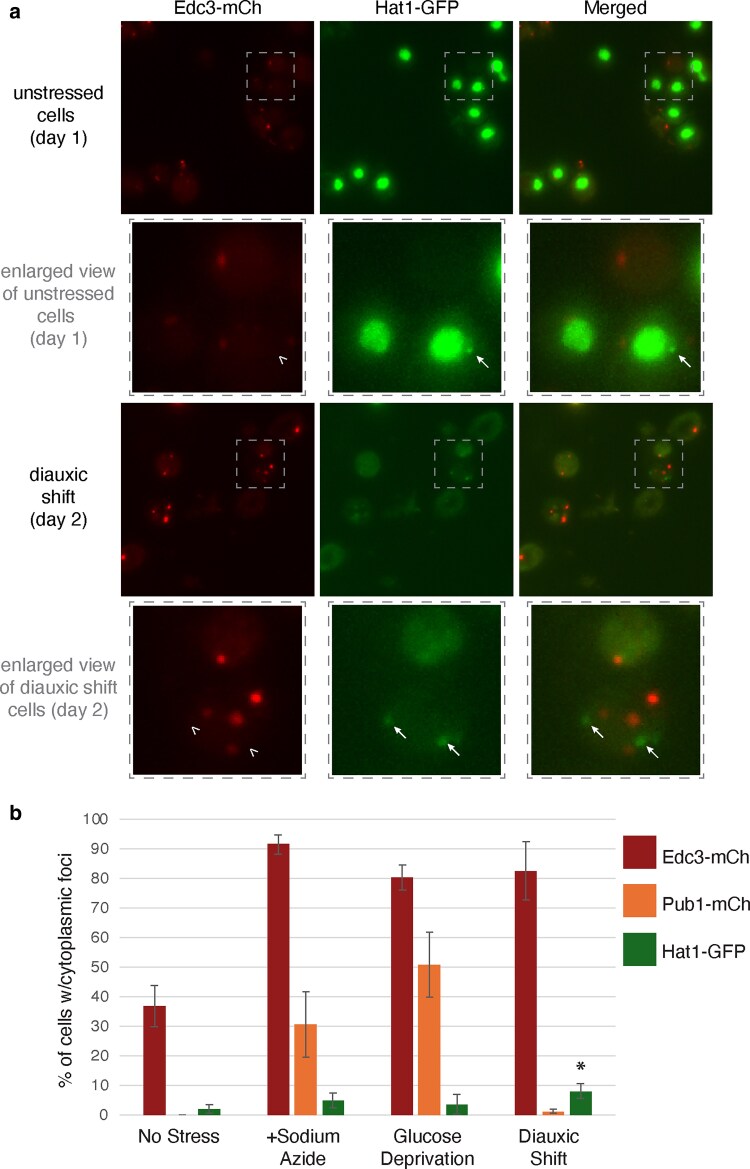
Hat1p is not present in P-bodies or stress granules, but does aggregate in rare uncharacterized cytoplasmic foci. Cells containing GFP inserted behind the chromosomal copy of *HAT1* (yAKH290) were transformed with a plasmid containing either Edc3p-mCh (pAKH96) to mark P-bodies (as shown in a) or Pub1p-mCh (pAKH94) to mark stress granules (shown in Supplementary Fig. 6 in Supplementary Materials). Cells in the logarithmic growth phase were imaged with and without stress. a) A sample microscopy image from unstressed and sodium azide-stressed (+NaAz) cells is shown with Edc3-mCh and Hat1-GFP. The cells marked with a dashed box in the first and third row are shown in a zoomed-in view just below the image. Hat1p-GFP forms rare cytoplasmic foci (marked by white arrows), so a cell containing Hat1 foci is intentionally shown, despite their rarity. The arrowheads mark where the Hat1-GFP foci would be on the mCherry image, demonstrating that there is no Edc3-mCh foci co-localizing with Hat1-GFP. b) Quantitation of all tested conditions, showing percentage of cells that contain cytoplasmic foci formed by Edc3-mCh (P-body marker), Pub1(stress granule marker), or Hat1-GFP. Each average is from at least 3 independent experiments and at least 300 cells per replicate. Error bars show the SD. The asterisk represents *P* < 0.05 from an unpaired two-tailed *t*-test comparing the percentage of cells with Hat1-GFP foci in diauxic shift vs no stress. Sample images, with a P-body or a stress granule marker, of conditions not shown in (a) are shown in Supplementary Fig. 6 in Supplementary Materials.

Given the effect of *HAT1* on affecting P-bodies and stress granules, we tested whether Hat1p can accumulate in these cytoplasmic granules. Hat1p is concentrated in the nucleus with diffuse cytoplasmic localization (Poveda et al. 2004). We tagged the genomic copy of *HAT1* with GFP and examined its localization under stress compared to known P-body (Edc3p-mCh) and stress granule (Pub1p-mCh) markers. We assessed localization in the absence of stress, after sodium azide or glucose deprivation treatment, and after 2 d of growth, where cells enter diauxic shift (Fig. 6 and Supplementary Fig. 6). The percentage of cells with P-bodies increases in all 3 stresses, while the percentage of cells with stress granules increases after sodium azide treatment and glucose deprivation, but not during diauxic shift. Hat1p-GFP localizes to cytoplasmic foci in a small percentage of cells, with a statistically significant increase during diauxic shift, where 8% of cells have Hat1p-GFP foci, compared to 2% in nonstressed, logarithmically growing cells (Fig. 6b). Hat1p-GFP did not localize with Edc3p or Pub1p foci in any condition (sample images shown in Fig. 6 and Supplementary Fig. 6), suggesting that Hat1p does not accumulate in P-bodies or stress granules, at least not in levels detectable by microscopy under these conditions. Hat1p-GFP does get more diffusely localized across the cytoplasm during diauxic shift, compared to the strongly nuclear localization seen in the other conditions (Fig. 6a).

### Identifying genetic interactions between *DED1* and other KATs/KDACs

We hypothesize that the genetic interaction between *HAT1* and *DED1* suggests a role for Hat1p in cytoplasmic mRNA regulation, but not via a direct interaction with Ded1p. As overexpression of *DED1* interferes with translation by accumulating mRNPs in large stress granules (Hilliker et al. 2011), these mutant yeast already grow poorly (Fig. 1), and are likely sensitive to additional changes in translation and/or mRNA granules. To test whether the genetic interaction is unique to *HAT1*, we used *DED1* overexpression as a genetic tool to test for genetic interactions with other confirmed and putative KATs by screening available null mutations in the catalytic components of nonessential KATs (Downey and Baetz 2016).

**Fig. 7.**
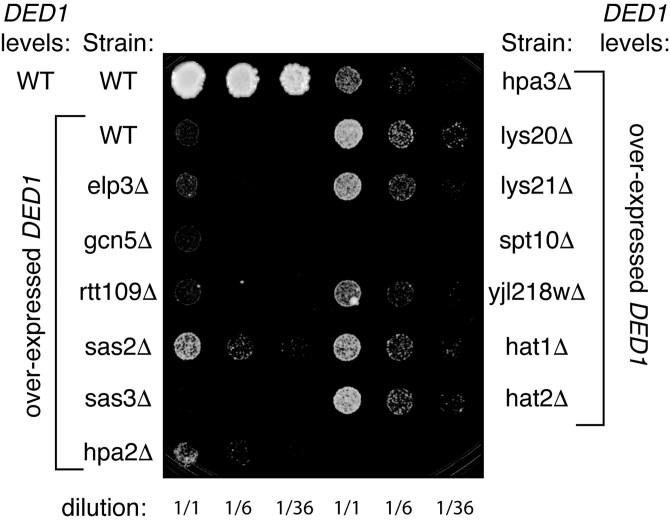
*DED1* has a genetic interaction with several lysine acetyltransferases. Strains deleted for one nonessential lysine acetyltransferase catalytic subunit (KAT; Supplementary Table 1 in Supplementary Materials) were transformed either with a plasmid lacking *DED1* (marked as WT under *DED1* levels; pRS423), to maintain endogenous levels of Ded1p, or with a plasmid overexpressing *DED1* from a galactose-inducible promoter (pAKH201). Strains were plated at the same concentration on selective media containing 2% galactose. Cells shown were grown for 6 d at 30 °C. All of the null strains grow like wild-type on selective media with 2% glucose, regardless of the plasmid transformed; these null KAT strains, when transformed with the empty vector (pRS423), grow like wild-type on selective media with 2% galactose (Supplementary Fig. 7). Note that *YJL218W* is a putative acetyltransferase.

Among the 9 confirmed nonessential acetyltransferase catalytic subunits, 3 null mutants suppressed the *DED1* overexpression growth defect (*sas2Δ*, *hpa3Δ,* and *hat1Δ*), 2 null mutants enhanced the growth defect (*sas3Δ*, *spt10Δ*), and the 4 remaining null mutants had no reproducible effect (*elp3Δ*, *gcn5Δ*, *rtt109Δ*, *hpa2Δ*; Fig. 7). None of these deletion strains had growth defects on their own, except for *gcn5Δ*, which has a slight growth defect on galactose media (Supplementary Fig. 7 in Supplementary Materials).

We find that deletion of either *HAT1* or its co-factor *HAT2* is a weak suppressor of the *DED1* overexpression growth defect (Fig. 7). Recall that overexpression of *HAT1* also suppresses the *DED1* overexpression growth defect (Fig. 1). These results can be reconciled if Hat1p affects the acetylation of multiple proteins involved in different steps of translation and mRNA granule accumulation. We have observed a similar phenomenon for *DED1*, where both loss-of-function and overexpression alleles of *DED1* cause a loss in translation, in that case, likely reflecting that each *ded1* allele damages a different function of Ded1p (Hilliker et al. 2011).

We do not see any obvious patterns in the literature demonstrating characteristics that all of the suppressors share and that differentiate them from nonsuppressors. These KATs target a variety of different histone and nonhistone targets. They are known to affect various chromatin-related functions, like transcription, telomere maintenance, and DNA repair. But we find no obvious pattern in why some KATs suppress and others do not.

We screened nulls of a few nonessential enzymes that consumed acetyl-CoA in other ways aside from lysine acetylation (*lys20Δ, lys21Δ, yjl218wΔ*). Intriguingly, all 3 of these null strains suppressed the *DED1* overexpression growth defect (Fig. 7). Lys20p and Lys21p have weak in vitro KAT activity, but are better known for their role in condensing acetyl-CoA to form homocitrate, the rate-limiting step in lysine biosynthesis (Scott and Pillus 2010). The target and function of *YJL218W* is unknown, but it has homology to bacterial galactoside *O*-acetyltransferases, which use acetyl-CoA to modify sugars (Karpichev and Small 1998). Consistent with a broader sensitivity to acetyl-CoA, Hpa2p and Hpa3p, while sharing high sequence homology, have distinctions in their function and in our screen. *HPA3*, when deleted, suppresses the *DED1* overexpression phenotype, but deletion of *HPA2* does not (Fig. 7). *HPA3* can acetylate a wide range of D-amino acids, but *HPA2* does not.

**Fig. 8.**
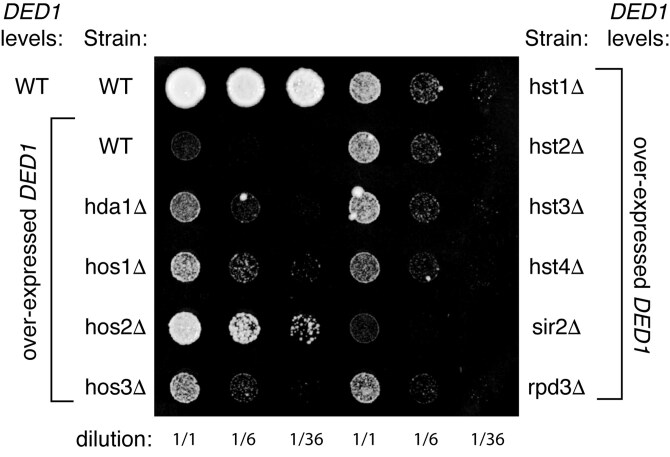
*DED1* has a genetic interaction with several lysine deacetylases. Strains deleted for one nonessential lysine deacetylase (KDAC; Supplementary Table 1 in Supplementary Materials) were transformed and plated on minimal media containing 2% galactose as in Fig. 7. Controls grown on glucose-containing media and controls with endogenous levels of Ded1p are shown in Supplementary Fig. 8 in Supplementary Materials.

If *DED1* overexpression is sensitizing cells to either lysine acetylation or acetyl CoA levels, then we might expect genetic interactions with lysine deacetylases (KDACs) as well. We tested whether confirmed KDACs (Downey and Baetz 2016) could suppress the *DED1* overexpression growth defect. All of the tested KDAC deletions, with the exception of *sir2Δ,* suppressed the *DED1* overexpression growth defect to varying extents (Fig. 8) but had no significant growth differences in yeast with endogenous levels of *DED1* (Supplementary Fig. 8 in Supplementary Materials). Some of these KDACs are implicated in maintaining the boundaries of telomeres (Rpd3p and Hos2p; Zhou et al. 2009; Ehrentraut et al. 2010) or promoting telomere silencing (Hst1p, Hst3p, Hst4p; Brachmann et al. 1995), while Hst2p antagonizes telomere silencing (Perrod et al. 2001; Keogh et al. 2005; Zhou et al. 2009). The opposing effects of these factors are consistent with a complex and indirect relationship between acetylation levels and Ded1p.

With the numerous genetic interactions that we observe between acetyltransferases and deacetylases, there may be unique mechanisms at play among suppressors. For example, some of the KDAC suppressors are involved in silencing genes related to translation. Hos1p contributes to silencing near tRNA genes (Good et al. 2013). Microarray deacetylation maps show that Hos1p and Hos2p preferentially deacetylate near rRNA and ribosomal protein genes (Robyr et al. 2002). Thus, their deletion might increase transcription of ribosomal components and suppress the overexpression of *DED1* by providing more translation machinery to counteract *DED1-mediated* mRNA storage. Future mechanistic studies would be needed to determine why Ded1 function is sensitive to acetylation.

Consistent with our broad findings, a recent synthetic genetic array (SGA) screen found genetic interactions between a *ded1* loss-of-function allele and multiple acetyltransferases. In this screen, yeast with a C-terminal truncation of *ded1* were crossed to a yeast library of strains with a nonessential gene deleted and tested for growth in the presence of the mTOR inhibitor rapamycin (Carey et al. 2023). As our screen used a *DED1* overexpression phenotype, rather than a loss-of-function mutation, we expect the gene deletion of a KAT to have the opposite effect on the *ded1* C-terminal truncation, compared to *DED1* overexpression in our screen. Indeed, where we identified *spt10Δ* as 1 of 2 KATs that enhanced the *DED1* overexpression phenotype, the SGA screen identified *spt10Δ* as a suppressor of truncated *ded1*. Similarly, we identified deletion of KATs *LYS20, HPA3,* and *YJL218W* as suppressors of the *DED1* overexpression phenotype, whereas the SGA screen identified the null mutants as enhancers of the *ded1* truncation. Our screen is consistent with the genetic observations in Carey et al. (2023), but also identifies new KATs and KDACs that have a genetic interaction with *DED1.*

## Conclusions

Through an open-ended overexpression screen, we found that overexpression of the acetyltransferase, *HAT1*, suppresses the growth defect conferred by excess Ded1p. We did not find evidence that Ded1p is directly acetylated, suggesting that the genetic interaction may reflect a more indirect functional relationship.

We found that cells lacking *HAT1* showed an increase in P-bodies under short-term glucose deprivation and sodium azide stress, suggesting that wild-type *HAT1* may antagonize P-body formation. The *hat1* null mutant shows a significant growth advantage, compared to wild-type, under long-term glucose deprivation, as cells move through diauxic shift and into the stationary phase. These data are the first tractable phenotype of the *hat1* null and will help with future genetic studies of *HAT1.* This growth advantage correlates with increased accumulation of stress granules.

Consistent with a more indirect relationship between *HAT1* and *DED1*, we have identified genetic interactions between *DED1* overexpression and 5 additional acetyltransferases and 9 KDACs, suggesting a link between mRNA regulation and either lysine acetylation or some other effect on cellular levels of acetyl CoA. There are other known links between KATs and KADCs affecting translation or cytoplasmic RNA granules, as reviewed above. Our data increase the known connections between mRNA regulation and acetylation, increasing the number of KATs and KDACs that might influence cytoplasmic mRNA regulation.

Future work could address several possible mechanisms of why *DED1* is affected by acetyltransferase or KDAC genes. First, it is increasingly clear that many KATs and KDACs have nonhistone, cytoplasmic targets. Some of these KATs and KDACs that have genetic interactions with *DED1*, including Hat1p, show some cytoplasmic localization. While we do not detect acetylation of Ded1p, proteomic studies indicate that a variety of translation initiation factors are acetylated (Henriksen et al. 2012), including eIF4E and eIF4A proteins, which interact with Ded1p (Gao et al. 2016; Gulay et al. 2020). An acetylation-mimicking mutation in *PAB1* decreases glucose deprivation stress granules, suggesting that acetylation of Pab1p would antagonize stress granules (Sivananthan et al. 2023). Second, Ded1p might alter the activity of certain KATs or KDACs. Overexpression of DDX3, the human homolog of Ded1p, co-purifies with CBP-p300, an acetyltransferase known to acetylate transcription factor HNF4. Overexpression of DDX3 correlates with an increase in HNF4 acetylation in vivo and increased transcription of an HNF4 gene target, MTP (Tsai et al. 2017), suggesting that DDX3 promotes CBP-p300 function. Third, as some suppressors code for enzymes that use acetyl-CoA as substrates for reactions other than lysine acetylation, the numerous genetic interactions we identify may reflect a broader connection between translation regulation and stores of cellular energy. Levels of acetyl-CoA are directly correlated with the energy status of the cell, as it is a byproduct of glycolysis and pyruvate processing (Galdieri et al. 2014). Translation is one of the most energy-intensive processes in the cell. Many stressors that deplete energy stores cause a near global decrease in translation and a concomitant increase in cytoplasmic granules containing repressed mRNAs. Stress granule formation during glucose deprivation is antagonized by high levels of acetate in media, suggesting that something that affects granule accumulation is sensitive to acetyl CoA levels (Rollins et al. 2017). Future studies are needed to explore the possible mechanisms of how lysine acetylation and/or acetyl-CoA levels impact translation and mRNA storage.

## Supplementary Material

jkaf307_Supplementary_Data

## Data Availability

Strains and plasmids (listed in Supplementary Tables 1 and 2, Supplementary materials) are available upon request from the corresponding author. The authors affirm that all data necessary for confirming the conclusions of this article are represented fully within the article and its tables and figures. Supplemental material available at G3 online.
