# Supplementary material for: A genetic interaction between DED1 and HAT1 in Saccharomyces cerevisiae reveals a role for Hat1p in cytoplasmic RNA granule accumulation
Source: G3 (Bethesda). 2025 Dec 19;16(3):jkaf307. doi: 10.1093/g3journal/jkaf307 (PMC12958820; doi:10.1093/g3journal/jkaf307)
Supplement: jkaf307_Supplementary_Data [file jkaf307_supplementary_data.pdf]

Supplementary Materials

Table of Contents	Page
Supplementary Table 1	2
Supplementary Table 2	3-4
Supplementary Table 3	5
Supplementary Figure 1	6
Supplementary Figure 2	7
Supplementary Figure 3	8
Supplementary Figure 4	9
Supplementary Figure 5	10
Supplementary Figure 6	11-12
Supplementary Figure 7	13-14
Supplementary Figure 8	15
Supplementary Methods	16
Supplementary Literature Cited	17

Table S1. Yeast strains used in study.

Strain	**Genotype**	**Reference or source**
BY4741	MAT**a** *ura3Δ0, leu2Δ0, his3Δ1, met15Δ0*	ATCC 4040002
yAKH174/ yRP2799	MAT**a** *ura3Δ0, leu2Δ0, lys2Δ0, his3Δ1, met15Δ0, ded1::KANMX* pRP1560	(Hilliker et al. 2011)
yAKH201	MAT**a** *ura3Δ0, leu2Δ0, his3Δ1, met15Δ0 hat1::KanMX*	(Winzeler et al. 1999)
yAKH210	MAT**a** *ura3Δ0, leu2Δ0, his3Δ1, met15Δ0 edc3::KanMX*	(Winzeler et al. 1999)
yRP840	MAT**a** *ura3-52, leu2-3,112, his4-539, trp1 cup1::LEU2/PGK1pG/MFA2pG*	(Hatfield et al. 1996)
yAKH290	MAT**a** *ura3-52, leu2-3,112, his4-539, trp1 cup1::LEU2/PGK1pG/MFA2pG HAT1-GFP:: KanMX6 (parent- yRP840)*	This study
yRP2337	MAT**a** *leu2-3,112 trp1 ura3-52 lys2 cup1::LEU2/PGK1pG/MFA2pG lsm4ΔC::NEO*	(Decker et al. 2007)
yRP2338	MAT**a** *leu2-3,112 trp1 ura3-52 his4-539 cup1::LEU2/PGK1pG/MFA2pG lsm4ΔC::NEO edc3:: NEO*	(Decker et al. 2007)
KAT/KDAC nulls in BY4741 background	MAT**a** *ura3Δ0, leu2Δ0, his3Δ1, met15Δ0 gene of interest::KanMX*	(Winzeler et al. 1999)
other PB/SG nulls	MAT**a** *ura3Δ0, leu2Δ0, his3Δ1, met15Δ0 gene of interest::KanMX*	(Winzeler et al. 1999)

Table S2. Plasmids used in study.

Plasmid	Description	Vector	Insert	Source
pRP1367	pFA6a-GFP(S65T)-KanMx	pFA6a	GFP(S65T)-KanMx	(Bähler et al. 1998)
pAKH29	pRS413	pRS413 (*HIS3*/*CEN*)	none	(Sikorski and Hieter 1989)
pAKH32/ pRP1555	*DED1* in pRS413	pRS413 (*HIS3*/*CEN*)	*DED1* under endogenous promoter	(Hilliker et al. 2011)
pRS423	pRS423	pRS423 (*HIS3/2μ*)	none	(Sikorski and Hieter 1989)
pAKH51/ pRP1657	*Pab1-GFP/ Edc3-mCh*	YCPLAC33-derived (*URA3/CEN*)	*EDC3* promoter and CDS-*mCherry*; *PAB1* promoter and CDS-*GFP*	(Buchan et al. 2008)
pAKH94/ pRP1661	*Pub1-mCh*	YCPLAC33-derived (*URA3/CEN*)	*PUB1* promoter and CDS-*mCherry*	(Buchan et al. 2008)
pAKH96/ pRP1574	*Edc3-mCh*	YCPLAC33-derived (*URA3/CEN*)	*EDC3* promoter and CDS-*mCherry*	(Buchan et al. 2008)
pAKH201/ pRP2086	*GAL-DED1*	pRS423 (*HIS3/2μ*)	*GAL-DED1-6xHIS-HA-protein A*	(Hilliker et al. 2011)
YEP24	YEP24	YEP24 (*URA3/2μ*)	none	ATCC 37051
pAKH254	Library plasmid containing *HAT1*	YEP24 (*URA3/2μ*)	Chromosome XVI 553662-559121	(Carlson and Botstein 1982)
pAKH257	*HAT1* in YEP24	YEP24 (*URA3/2μ*)	*HAT1 cds +/- 500 nt*	This study
pAKH258	*GAL-HAT1* in BG1766	BG1766 (*URA3/2μ*)	*GAL-HAT1-6xHIS-HA-protein A*	(Gelperin et al. 2005)
pAKH290	*hat1-G230A* in YEP24	YEP24 (*URA3/2μ*)	*hat1-G230A cds +/- 500 nt*	This study
pAKH291	*hat1-I222A* in YEP24	YEP24 (*URA3/2μ*)	*hat1-I222A cds +/- 500 nt*	This study

pAKH292	*hat1-E255A* in YEP24	YEP24 (*URA3/2μ*)	*hat1-E255A cds +/- 500 nt*	This study
pAKH306	BG1766	BG1766 (*URA3/2μ*)	none	(Gelperin et al. 2005)
pAKH319	*FLAG-DED1* in pRS413	pRS413 (*HIS3*/*CEN*)	pAKH32 with N-terminal FLAG tag added	This study
pAKH320	*DED1-FLAG* in pRS413	pRS413 (*HIS3*/*CEN*)	pAKH32 with C-terminal FLAG tag added	This study
pAKH721	*HAT1* in pRS423	pRS423 (*HIS3/2μ*)	*HAT1 cds +/- 500 nt*	This study
pAKH834	*HAT1-myc* in YEP24	YEP24 (*URA3/2μ*)	pAKH257 with C-terminal myc tag added	This study

Table S3. Primers sequences for plasmid and strain construction.

oAKH183	5’ cca cta tcg act acg cga tca tgg cga cca cac ccg tcc ggg ccc ctc gat caa ata aag ata ggg gat c
oAKH184	5’ cac ctg tgg cgc cgg tga tgc cgg cca cga tgc gtc cgg cgg ggc ccg tgt gga gta agt cat caa aac
oAKH299	5’ tta tag gcg tat tat aga gtc gat aaa taa atc tca agg tcg gat ccc cgg gtt aat ta
oAKH300	5’ gtt aaa caa ata aat atg tta tta tat att taa taa aca gga att cga gct cgt tta aac
oAKH679	5’ ggc gga ggt gga ggg gaa caa aag cta atc tcc gag gaa gac ttg taa ctg ttt att aaa tat ata ata aca tat tta ttt gtt taa caa gcc
oAKH680	5’ taa ctg ttt att aaa tat ata ata aca tat tta ttt gtt taa caa gcc
oAKH681	5’ caa gtc ttc ctc gga gat tag ctt ttg ttc ccc tcc acc tcc gcc acc ttg aga ttt att tat cga ctc tat aat acg
oAKH682	5’ acc ttg aga ttt att tat cga ctc tat aat acg

Supplemental Figures

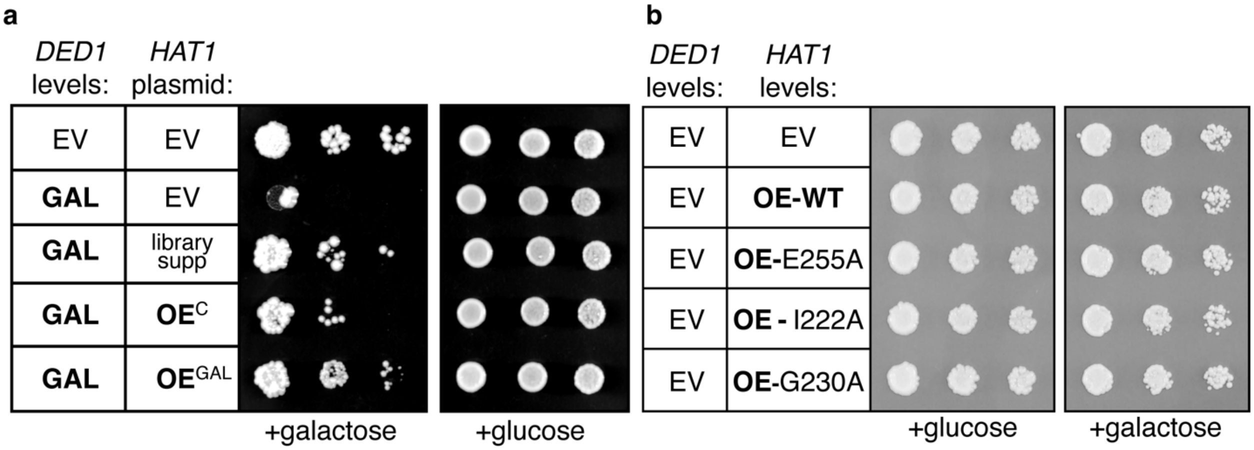

Fig. S1 The growth defect conferred by overexpression of *DED1* can be suppressed by overexpression of *HAT1*. (A) Yeast strain BY4741 was transformed either with an empty vector (E; pRS423), a plasmid that could cause galactose inducible overexpression of *DED1* (OE; pAKH201). In addition, these strains were transformed with a plasmid that had either no insert (vector; YEP24), the original genomic suppressor identified in the screen (library sup; pAKH254), or *HAT1* gene cloned by itself into the same vector (OE^{C}; pAKH257), showing that constitutive overexpression of *HAT1* alone is sufficient for suppression. We also tested whether *HAT1* overexpression from a galactose inducible promoter (OE^{GAL}; pAKH258) could suppress the defect from overexpression of *DED1*. (B) Constitutive overexpression of wild-type or mutant *hat1* alleles do not impact the growth of yeast with endogenous (E) levels of Ded1p.

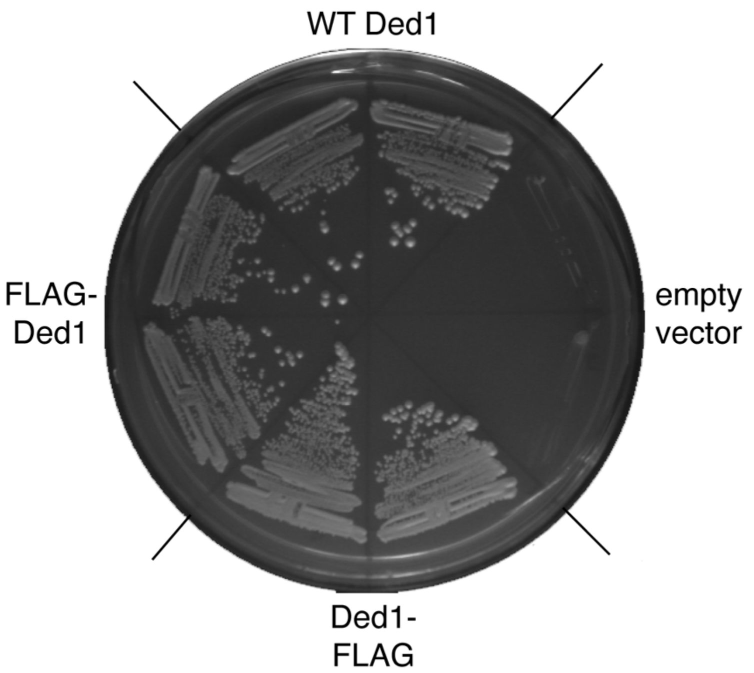

Fig. S2 Controls for *DED1* and *HAT1* constructs. (A) Both N terminal and C terminally tagged *DED1* can complement a *ded1Δ* null. Yeast strain yAKH174, which contains a chromosomal deletion of *ded1* and has wild-type *DED1* on a *URA3*-marked plasmid, was transformed with plasmids either containing no *DED1* (empty vector; pAKH29), wild-type, untagged *DED1* (WT; pAKH32), N-terminally FLAG-tagged *DED1* (*FLAG-DED1*; pAKH319), or C-terminally tagged *DED1* (*DED1-FLAG*; pAKH320). These transformants were streaked on media counter-selective for the *URA3*-marked plasmid (5-FOA). Cells with *FLAG-DED1* and *DED1-FLAG* grew as well as cells with wild-type *DED1* on 5-FOA, suggesting that the FLAG tag does not interfere with the essential function of Ded1p.

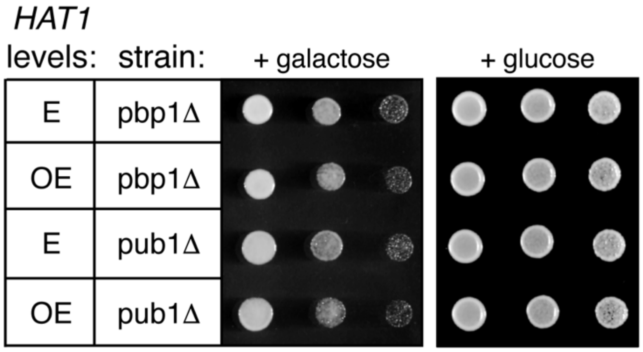

Fig. S3 Overexpression of *HAT1* does not affect the growth of stress granule assembly mutants. Strains were prepared and plated as described in Fig. 3.

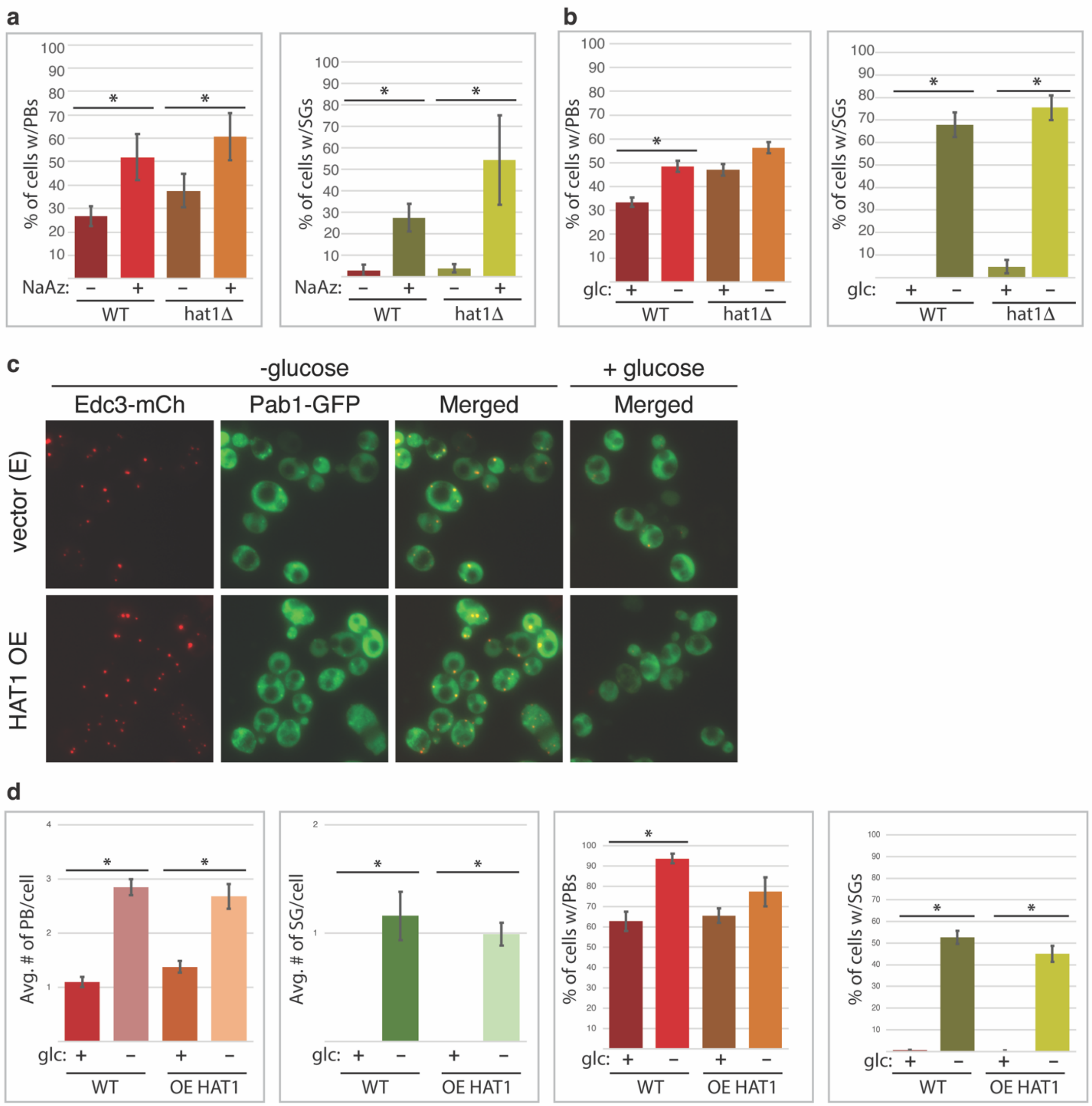

Fig. S4 Overexpression of *HAT1* lowers the percentage of cells that induce P-bodies during glucose deprivation. (A,B) Quantitation of percentage of cells with cytoplasmic granules from the experiment described in Fig. 4. Unpaired two-tailed t-tests evaluating each strain with versus without stress generated a p-value of less than 0.05 (as indicated by asterisk) except for yeast with *hat1*Δ with and without glucose. Any trends between percentage of cells with foci in WT versus *hat1*Δ strains under stress treatment was not statistically significant. (C) *HAT1* on a high-copy plasmid (OE; pAKH721) was transformed into yeast (BY4741) to constitutively overexpress the gene compared to strains with the empty vector (E; pRS423). Cytoplasmic granules were visualized and quantitated as in Fig. 4. (D) Quantitation of average granules per cell and percentage of cells with granules for the experiment shown in C. was completed as described in A. and in Fig. 4.

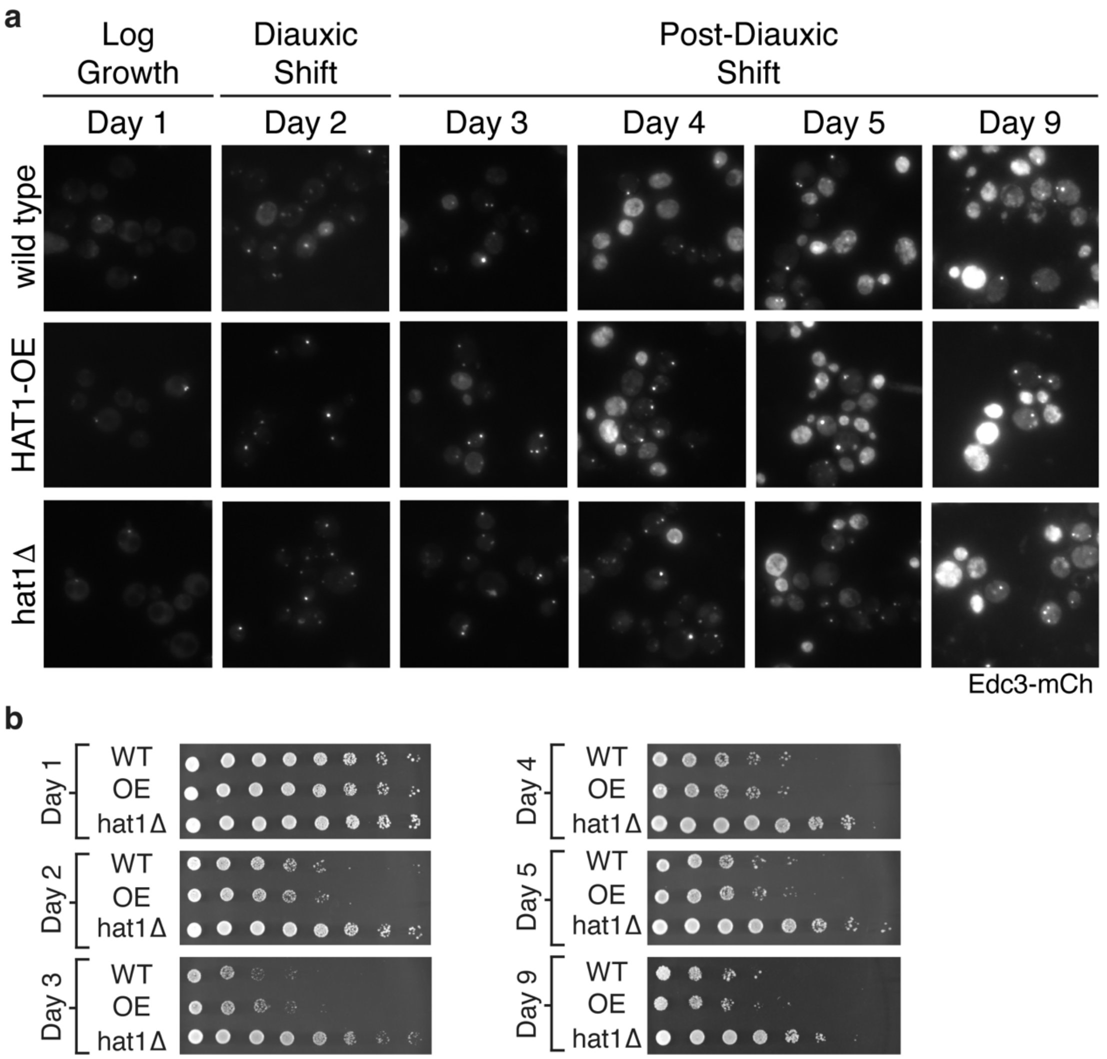

Fig. S5 Altered levels of Hat1p do not have an effect on P-bodies formed during diauxic shift and stationary phase. (A) P-body accumulation was tracked daily by Edc3p-mCh (pAKH51) localization in cells grown into stationary phase. Days 1-5 and 9 are shown. P-body formation persisted through the intervening days, 6-8. (B) These are the same plates shown in Fig. 5c after 2 days of incubation at 30°C.

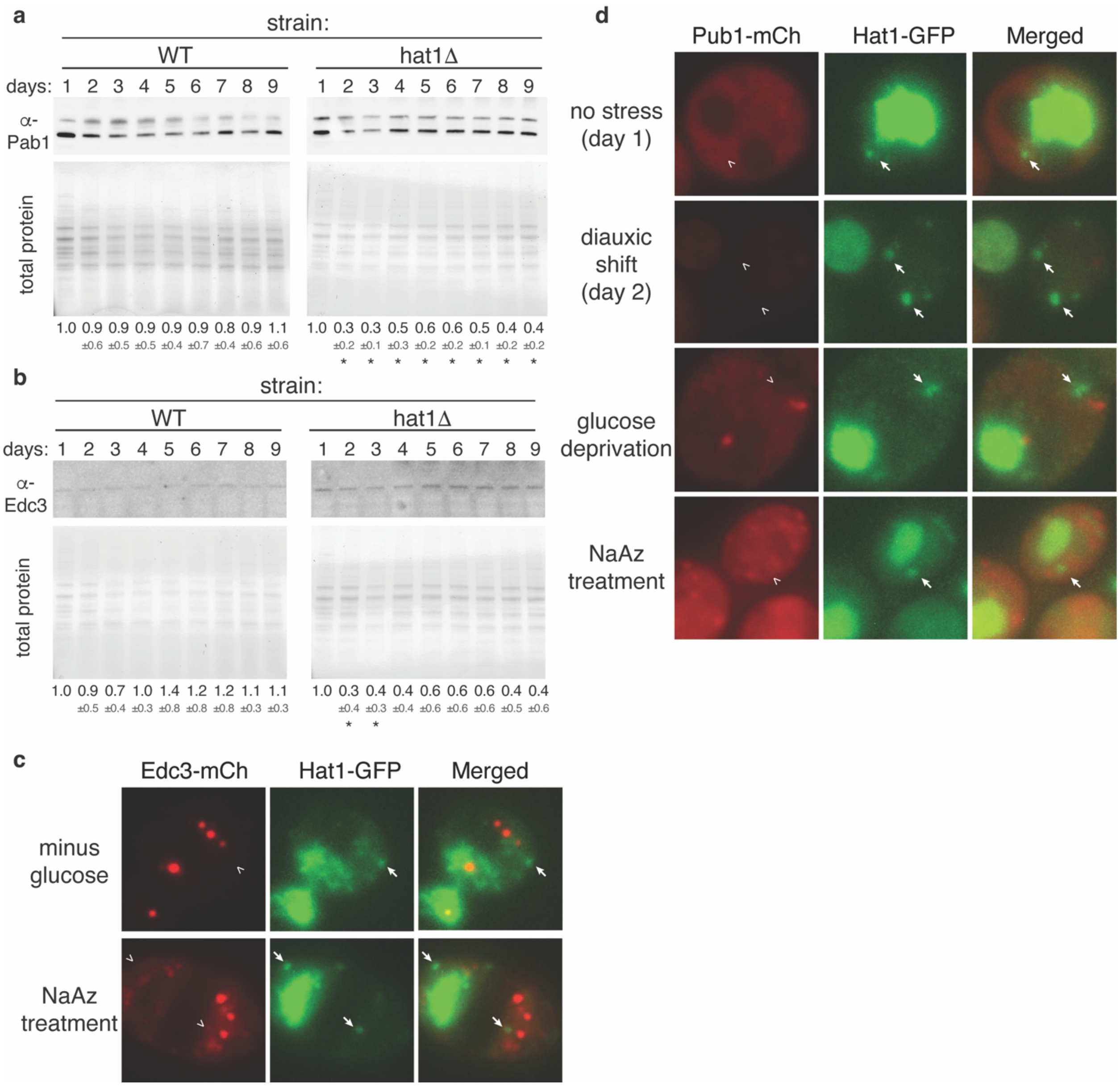

Fig. S6 Hat1p does not localize to P bodies or stress granules under a variety of stresses, but does stabilize Edc3p and Pab1p levels during diauxic shift. (a, b) Wild-type yeast (WT; BY4741 with pRS423) or yeast lacking *HAT1* (hat1Δ; yAKH201 with pRS423) were transformed with a plasmid containing Pab1p-GFP and Edc3p-mCh (pAKH51), as in Fig. 5. These strains were grown in selective media with 2% glucose for one to nine days at 30°C with shaking. Each day, the equivalent of 25 ml of culture at an optical density of 0.4 at 600 nm was pelleted and frozen in liquid nitrogen. Crude protein lysate was isolated from all pellets and total protein levels were assayed by Bradford assay. 30 ug of total protein was loaded onto 10% TGX Stain-Free Protein Gels (BioRad), which allowed visualization of total protein loaded on the gel. Blots were probed with anti-Pab1p (a) or anti-Edc3p (b) antibodies. The ratio of signal in Pab1p (or Edc3) bands to total protein levels were calculated and then normalized to the signal on day 1 of that same culture. See Supplemental Methods in Supplemental Materials for more information. Note that both endogenous and GFP-tagged Pab1p were visible, but only Edc3p-mCh was visible in our Western blots. This blot represents one of three trials, and the average (± standard deviation) of

the three ratios is shown. Any growth changes (compared to day 1) that were statistically significant are marked with an asterisk ($p < 0.05$; unpaired two-tailed t-test). (c) Close up images of rare cells that contain Hat1-GFP foci (marked with white arrow) compared to localization of P-body marker Edc3-mCh. The white arrow head marks where the Hat1-GFP foci would be in the mCherry image, to show that there is no Edc3-mCh foci at that position. A sample image from unstressed cells is shown in Fig. 6. (d) Close up images of rare cells that contain Hat1-GFP foci (marked with white arrow) compared to localization of stress granule marker Pub1-mCh. Samples of all conditions quantitated in Fig. 6 are shown.

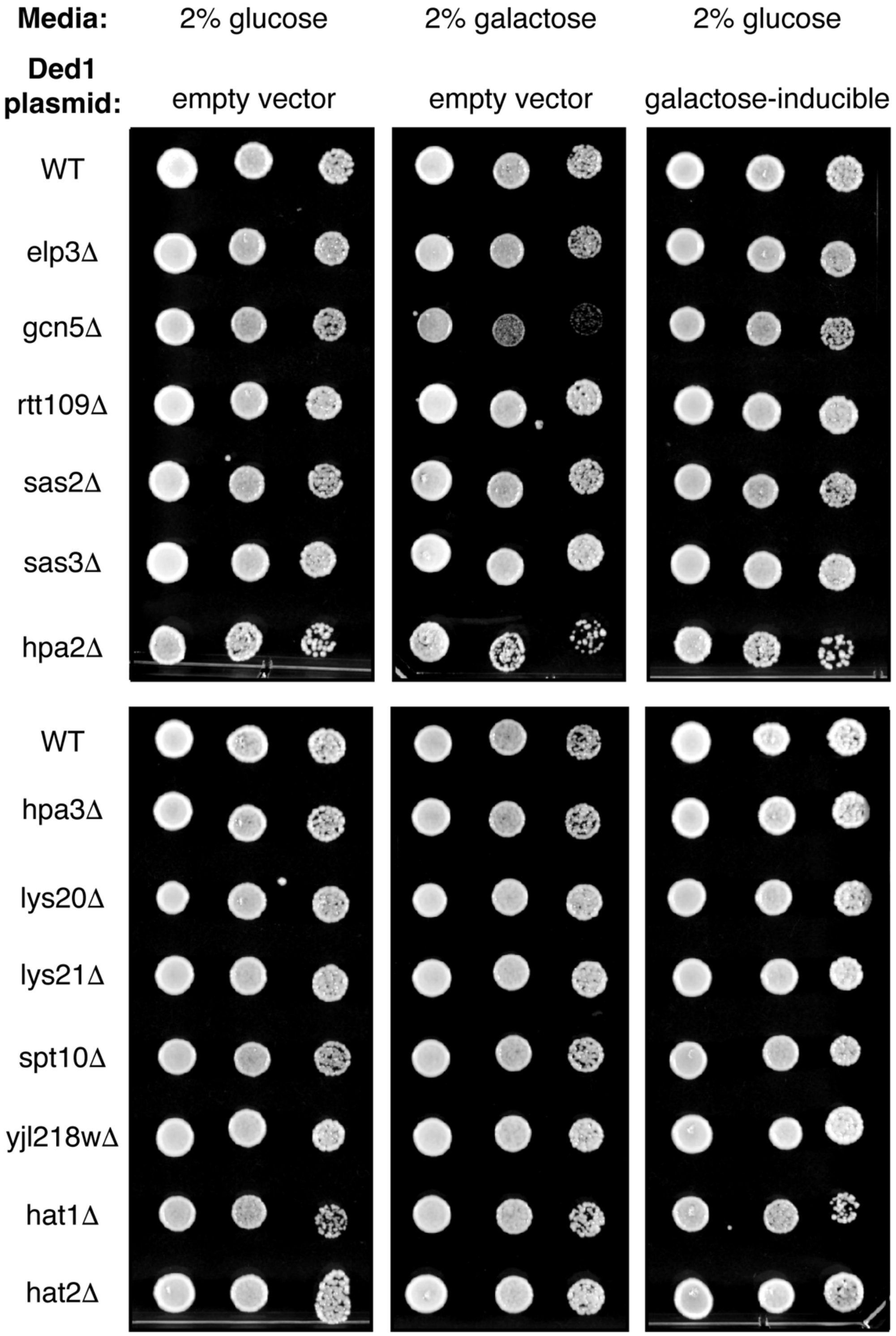

Fig. S7 Controls for genetic interaction tests between *DED1* and KATs. The deletion strains (listed at left) were transformed with either plasmid lacking *DED1* (left and middle images) or a plasmid with galactose-inducible *DED1* (right image), to test the effects of endogenous levels versus overexpression of *DED1*, as described in Fig. 7. Strains with endogenous *DED1* expression were grown on selective media containing either 2% glucose (left) or 2% galactose

(middle). Strains with overexpression of *DED1* were also grown on selective media containing either 2% glucose (right) or 2% galactose (Fig. 7). Yeast cells were spotted in 1/6 serial dilutions by multi-channel pipet.

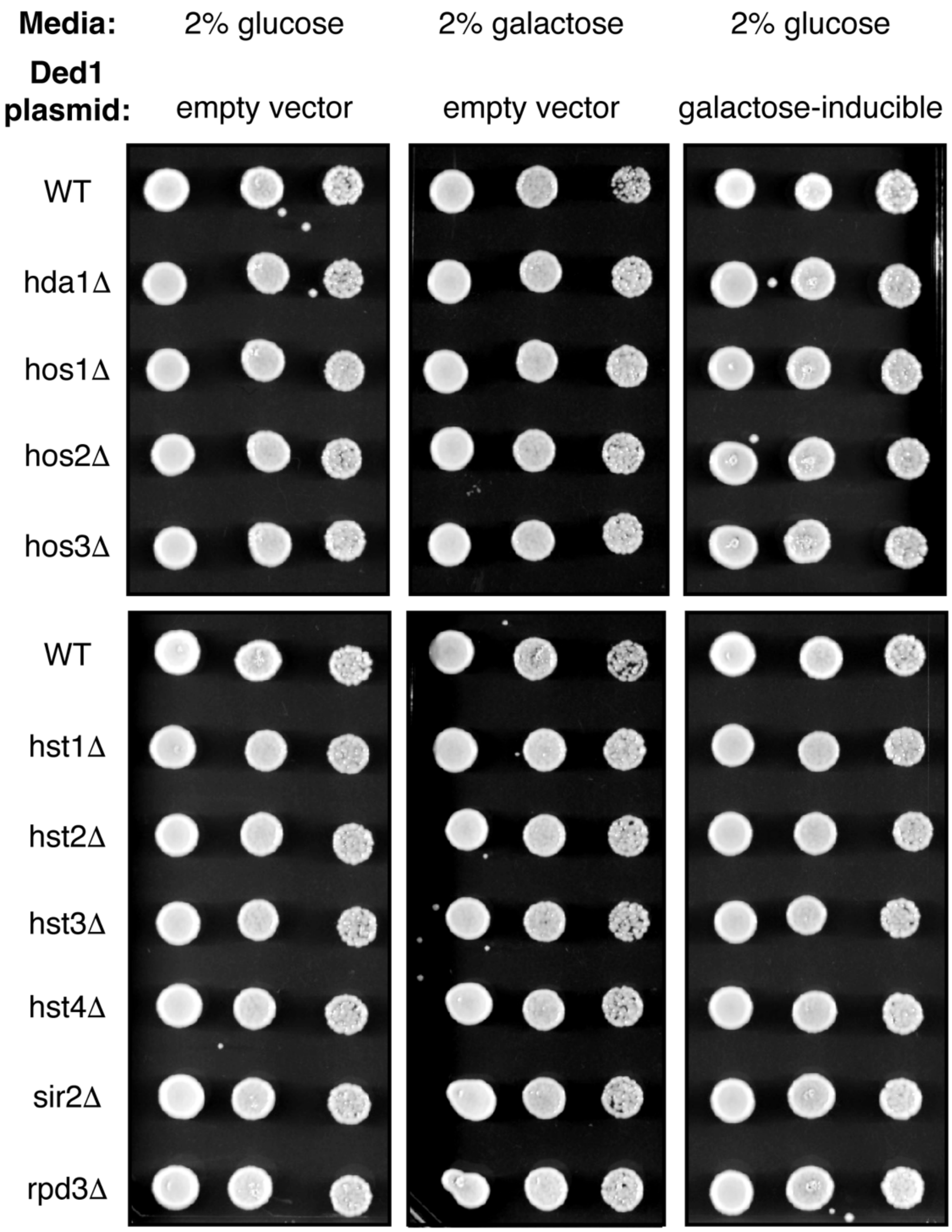

Fig. S8 Controls for genetic interaction tests between *DED1* and KATs. The deletion strains (listed at left) were transformed with either plasmid lacking *DED1* (left and middle images) or a plasmid with galactose-inducible *DED1* (right image), to test the effects of endogenous levels versus overexpression of *DED1*, as described in Fig. 8. Strains with endogenous *DED1* expression were grown on selective media containing either 2% glucose (left) or 2% galactose (middle). Strains with overexpression of *DED1* were also grown on selective media containing either 2% glucose (right) or 2% galactose (Fig. 8). Yeast cells were spotted in 1/6 serial dilutions by multi-channel pipet.

SUPPLEMENTARY METHODS:

Detecting levels of RNA granule proteins via western blot during extended growth

Cells were grown in selective media with 2% dextrose at 30°C. On each day (up to nine days), 25 ml of culture was pelleted and washed with media before freezing the pellet in liquid nitrogen. The first sample was collected while the culture was in mid-log growth. Pellets were resuspended in 2x pellet volume of 5 M urea and boiled at 100°C for 2 minutes. Cells were disrupted by vortexing for 5 minutes at 4°C with an equal volume of acid washed beads. 6x pellet volume of Solution A (125 mM Tris-HCl pH 6.8, 2% SDS) was added and samples were vortexed for 1 minute and then boiled for 2 minutes at 100°C. Crude protein lysate was removed from beads and clarified by spinning at top speed in a microfuge. Bradford assay was used to quantitate total protein levels. 30 µg of crude protein lysate was loaded on 10% Mini-Protean TGX Stain-Free Protein Gels (BioRad), which allow visualization of total protein levels. Levels of Edc3p and Pab1p were assayed by western blot with polyclonal antibodies raised in rabbits as in (Decker et al. 2007) for anti-Edc3p and as in (Buchan et al. 2011) for anti-Pab1p. The intensity of the Edc3p or Pab1p bands from the western blots and the intensity of the total protein levels from the equivalent lane on the gel was quantitated in Image Lab (v. 6.1.0, BioRad). The ratio of Pab1p or Edc3p to total protein within the lane was calculated. All ratios were normalized to the signal of wildtype cells on day 1 of growth.

Literature cited

Bähler J, Wu J-Q, Longtine MS, Shah NG, Mckenzie III A, Steever AB, Wach A, Philippsen P, Pringle JR. 1998. Heterologous modules for efficient and versatile PCR-based gene targeting in Schizosaccharomyces pombe. *YEAST*. 14(10):943–951. https://doi.org/10.1002/(SICI)1097-0061(199807)14:10%253C943::AID-YEA292%253E3.0.CO;2-Y.

Buchan JR, Muhlrad D, Parker R. 2008. P bodies promote stress granule assembly in Saccharomyces cerevisiae. *J CELL BIOL*. 183(3):441–455. https://doi.org/10.1083/jcb.200807043.

Buchan JR, Yoon JH, Parker R. 2011. Stress-specific composition, assembly and kinetics of stress granules in Saccharomyces cerevisiae. *J CELL SCI*. 124(Pt 2):228–239. https://doi.org/10.1242/jcs.078444.

Carlson M, Botstein D. 1982. Two differentially regulated mRNAs with different 5′ ends encode secreted and intracellular forms of yeast invertase. *CELL*. 28(1):145–154. https://doi.org/10.1016/0092-8674(82)90384-1.

Decker CJ, Teixeira D, Parker R. 2007. Edc3p and a glutamine/asparagine-rich domain of Lsm4p function in processing body assembly in Saccharomyces cerevisiae. *J CELL BIOL*. 179(3):437–449. https://doi.org/10.1083/jcb.200704147.

Gelperin DM et al. 2005. Biochemical and genetic analysis of the yeast proteome with a movable ORF collection. *GENES DEV*. 19(23):2816–2826. https://doi.org/10.1101/gad.1362105.

Hatfield L, Beelman CA, Stevens A, Parker R. 1996. Mutations in trans-acting factors affecting mRNA decapping in Saccharomyces cerevisiae. *MOL CELL BIOL*. 16(10):5830–5838. https://doi.org/10.1128/MCB.16.10.5830.

Hilliker A, Gao Z, Jankowsky E, Parker R. 2011. The DEAD-box protein Ded1 modulates translation by the formation and resolution of an eIF4F-mRNA complex. *MOL CELL*. 43(6):962–972. https://doi.org/10.1016/j.molcel.2011.08.008.

Sikorski RS, Hieter P. 1989. A system of shuttle vectors and yeast host strains designed for efficient manipulation of DNA in Saccharomyces cerevisiae. *GENETICS*. 122(1):19–27. https://doi.org/10.1093/genetics/122.1.19.

Winzeler EA et al. 1999. Functional characterization of the S. cerevisiae genome by gene deletion and parallel analysis. *SCIENCE*. 285(5429):901–906. https://doi.org/10.1126/science.285.5429.901.
